# Risk factors associated with post-tuberculosis sequelae: a systematic review and meta-analysis

**DOI:** 10.1016/j.eclinm.2024.102898

**Published:** 2024-10-21

**Authors:** Temesgen Yihunie Akalu, Archie C.A. Clements, Alemneh Mekuriaw Liyew, Beth Gilmour, Megan B. Murray, Kefyalew Addis Alene

**Affiliations:** a School of Population Health, Faculty of Health Sciences, Curtin University, Perth, Western Australia, Australia; b Geospital and Tuberculosis Research Team, Telethon Kids Institute, Perth, Western Australia, Australia; c Institute of Public Health, College of Medicine and Health Sciences, University of Gondar, Gondar, Ethiopia; d School of Biological Sciences, Queen’s University of Belfast, United Kingdom; e Department of Global Health and Social Medicine, Harvard Medical School, Boston, MA, USA

**Keywords:** Tuberculosis, Physical sequelae, Systematic review, And meta-analysis

## Abstract

**Background:**

Post-tuberculosis (TB) sequelae present a significant challenge in the management of TB survivors, often leading to persistent health issues even after successful treatment. Identifying risk factors associated with post-TB sequelae is important for improving outcomes and quality of life of TB survivors. This systematic review and meta-analysis aims to identify risk factors associated with long-term physical sequelae among TB survivors.

**Methods:**

We systematically searched Medline, Embase, PROQUEST, and Scopus for studies on long-term physical sequelae among TB survivors up to December 12, 2023. The primary outcome of interest was to quantify risk factors of long-term physical sequelae (i.e., respiratory, hepatic, hearing, neurological, visual, renal, and musculoskeletal sequelae). We included all forms of TB patients who experienced long-term physical sequelae. We used narrative synthesis for risk factors reported once and random-effect meta-analysis for primary outcomes with two or more studies. Findings were presented with odds ratios (OR) and 95% confidence intervals (CI). Publication bias was assessed using funnel plots and Egger regression, and heterogeneity was examined with a Galbraith radial plot. The protocol was registered on Prospero (CRD42021250909).

**Findings:**

A total of 73 articles from 28 countries representing 31,553 TB-treated patients were included in the narrative synthesis, with 64 of these studies included in the meta-analysis. Risk factors associated with post-TB lung sequelae include older age (OR = 1.62, 95% CI: 1.07–2.47), previous TB treatment history (OR = 3.43, 95% CI: 2.37–4.97), smoking (OR = 1.41, 95% CI: 1.09–1.83), alcohol consumption (OR = 1.84, 95% CI: 1.04–3.25), smear-positive pulmonary TB diagnosis (OR = 3.11, 95% CI: 1.77–6.44), and the presence of radiographic evidence of pulmonary lesions at the commencement of treatment (OR = 2.04, 95% CI: 1.07–3.87). Risk factors associated with post-TB liver injury included pre-existing hepatitis (OR = 2.41, 95% CI: 1.16–6.08), previous TB treatment (OR = 2.64, 95% CI: 1.22–6.67), hypo-albuminemia (OR = 2.10, 95% CI: 1.53–2.88), HIV co-infection (OR = 2.72, 95% CI: 1.66–4.46), and CD4 count <200 mm^3^ in HIV-infected individuals (OR = 2.03, 95%CI: 1.26–3.27). Risk factors associated with post-TB hearing loss include baseline hearing problems (OR = 1.72, 95% CI: 1.30–2.26), and HIV co-infection (OR = 3.02, 95% CI: 1.96–4.64).

**Interpretation:**

This systematic review and meta-analysis found that long-term physical post-TB sequelae including respiratory, hepatic, and hearing impairment were associated with a range of socio-demographic, behavioral, and clinical factors. Identification of these risk factors will help to identify patients who will benefit from interventions to reduce the burden of suffering from post-TB treatment.

**Funding:**

Healy Medical Research Raine Foundation, the Australian National Health and Medical Research Council, and 10.13039/501100001797Curtin University Higher Degree Research Scholarship fund the study.

**Research in context**

**Evidence before this study**

Existing evidence shows that people who survive TB face a considerable and under-recognized burden of morbidity and mortality after completion of treatment. We searched Medline, Embase, SCOPUS, and PROQUEST databases from the inception of each database to 12 December 2023, for papers published in English, using terms related to TB, sequelae, and risk factors. Our search found meta-analyses quantifying the burden of post-TB sequelae and some observational studies reporting risk factors associated with post-TB sequelae. However, collective evidence on the risk factors associated with post-TB sequelae is lacking, and there is no comprehensive systematic review that synthesizes the impacts of socio-demographic, behavioral, and clinical factors on post-TB sequelae.

**Added value of this study**

Our comprehensive systematic review and meta-analysis identified several socio-demographic, behavioral, and clinical factors that are associated with post-TB lung sequelae. The study identified important risk factors contributing to post-TB lung sequelae including older age, TB treatment history, smoking, and alcohol consumption. Risk factors such as low CD4 count in HIV-infected individuals, pre-existing hepatitis, and hypo-albuminemia also contributed to post-TB liver injury. HIV co-infection was also identified as a risk factor for post-TB hearing loss and liver injury. Identification of these important risk factors helps to inform evidence-based strategies for the prevention of post-TB sequelae.

**Implications of all the available evidence**

The findings of this systematic review and meta-analysis indicate the multifactorial nature of post-TB sequelae and demonstrate the importance of considering socio-demographic, behavioral, and clinical factors in post-TB care. Health practitioners can utilize this information to target high-risk populations, tailor post-TB care plans, and reduce the long-term burden of post-TB sequelae on affected individuals. Moreover, the study contributes to the holistic management of TB, extending beyond the initial treatment phase to address the enduring health challenges survivors face.

## Introduction

Globally, tuberculosis (TB) is a leading cause of mortality and morbidity responsible for an estimated 10.6 million incident cases and 1.3 million deaths in 2022.^1^^,^^2^ In 2019, it was estimated that TB was responsible for the loss of 66 million disability-adjusted life years (DALYs).^3^ Although antibiotic treatment can be successful in averting death, there is evidence of long-term sequelae and an elevated mortality risk among TB survivors.^4^, ^5^, ^6^ Historically, TB research efforts have focused on alleviating the acute phase of the disease. Although there has been recent consideration of the consequences during the post-acute phase, this area of study is still in its infancy.^7^^,^^8^ Current disease burden estimates and policy considerations exclude post-TB sequelae despite modeling suggesting that long-term consequences may account for 47% of the total DALY estimate.^9^

TB sequelae can arise from the disease itself or as a consequence of treatment, manifesting as structural, functional, or, infectious complications, or psychosocial morbidities.^10^ Although TB sequelae may occur during active disease or treatment, they can also manifest after treatment completion and bacteriological cure.^11^ Research shows an increasing risk of sequelae developing with higher levels of drug resistance,^12^ a pattern that may reflect the increasing toxicity of treatment regimens or delays in obtaining a clinical response with an appropriate regimen.

As pulmonary disease is the most common manifestation of TB, most medical post-TB sequelae relate to lung disease. However, due to TB’s ability to disseminate and the side effects of treatment, other organ systems may also be affected.^13^ Although significant research has been undertaken to evaluate the risk factors associated with the development of TB disease,^2^^,^^14^^,^^15^ little is known about the risk factors associated with long-term sequelae.^7^^,^^16^ Recent research has investigated interventions to prevent post-TB sequelae,^17^ making it critical to understand the risk factors associated with their development.^16^ Identifying risk factors for post-TB sequelae facilitates the creation of targeted interventions and more efficient allocation of healthcare resources.^18^ Additionally, it provides valuable insights for policymakers and clinicians, aiding in the prevention and early management of these complications.^19^

Previous studies have identified that post-TB sequelae affect all age groups and have provided several recommendations for their prevention and treatment. Management strategies, including pulmonary rehabilitation, counseling, health education, close monitoring, early diagnosis, and prompt treatment for post-TB sequelae, are crucial for reducing mortality and improving the quality of life among TB survivors. However, these strategies should be adapted to the context and available resources and tailored to high-risk groups.^19^^,^^20^

This study aims to address these needs by identifying the risk factors associated with long-term TB sequelae in all forms of TB patients (DS- and DR-TB combined, regardless of bacteriological results).

## Methods

This systematic review and meta-analysis was conducted according to the recent Preferred Reporting Items for Systematic Reviews and Meta-analysis (PRISMA) guidelines.^21^ The protocol was registered at PROSPERO, CRD42021250909.

### Search strategy

The search was conducted in four databases (Medline, EMBASE, Scopus, and PROQUEST) from database inception to 12 December 2023 with no restriction on the year of publication or geographic region. The search strategy was developed according to the Medical Subject Headings (MeSH) using a combination of keywords related to TB, sequelae, and risk factors. The detailed electronic search strategy is presented in Supplementary File S1. The search strategy was developed with the support of library technicians experienced in conducting systematic reviews in the medical field. Hand searches were also conducted from reference lists of included studies.

### Study selection strategies

After the search was completed, all individual studies were exported to Endnote, duplicates were removed and studies were exported to Rayyan for screening by title, abstract, and full text. Studies were included if they met the following criteria: investigated patients with TB and reported risk factors for long-term TB sequelae (e.g., lung sequelae, hearing sequelae, neurological sequelae, acute liver injury, renal sequelae, visual sequelae, and musculoskeletal sequelae). Post-TB sequelae were chosen over post-lung diseases to ensure a more comprehensive review, including sequelae from extrapulmonary TB cases. Interventional and observational studies such as case–control, cross-sectional, prospective, and retrospective cohort studies were included in the systematic review. Studies were excluded if they only reported the incidence or prevalence of post-TB sequelae, treatment outcome, or short-term reversible side effects. Non-English language articles, case reports, conference abstracts, editorial letters, and studies with incomplete information were also excluded. During the title and abstract review process, two reviewers (TYA and AML) selected articles for full-text review based on the inclusion and exclusion criteria. The same two authors (TYA and AML) conducted the full-text review. In case of disagreement between the two reviewers, differences were resolved by consensus.

### Outcomes of the study

The study population included patients with TB of any type (pulmonary, extrapulmonary, drug-susceptible, and drug-resistant TB) who were treated for TB and experienced long-term physical sequelae either during TB treatment or post-treatment. The primary goal of the study was to evaluate the risk factors associated with long-term TB sequelae that result from TB disease or its medications. The primary outcome was measured as an odds ratio (OR) with corresponding 95% confidence intervals (CI). World Bank data (2022–2023) were used to classify countries by income level for a sub-group analysis.^22^

### Data extraction

Data extraction was conducted by the two independent reviewers (TYA and AML) using a Microsoft Excel 19 spreadsheet (Microsoft, Redmond, Washington, USA). The data extraction tool was piloted using five individual papers and refined as necessary. The following information was extracted from the shortlisted studies: name of the primary author, year of publication, year of data collection, country of the study, study design, study setting, study population (children, adults, and both), characteristics of participants (mean/median age, proportion of male participants), sample size, type of TB (DS-TB or DR-TB), type of TB treatment, timing of sequelae (during TB treatment or after TB treatment), duration of treatment, comorbidities (diabetes mellitus (DM) and HIV), and type of post-TB sequelae, and risk factors for post-TB sequelae.

### Quality assessment and risk of bias assessment

The quality of the included studies was assessed using the Newcastle Ottawa Scale (NOS) for observational studies. The quality evaluation was undertaken by two independent reviewers (TYA and AML) and in the case of discrepancy, a decision was reached by consensus. The NOS assessment scale generates a score from 0 to 9, and the subsequent scores were then categorized into low-quality (0–4 points), moderate quality (5–7 points), and high-quality studies (8–9 points).

### Statistical analysis

Initially, narrative synthesis was conducted for each primary outcome. When sufficient data were available (>2 studies per outcome of interest) a quantitative meta-analysis was performed using STATA version 17 software. Odds ratios (OR) and corresponding confidence intervals were reported and used to pool the effect size for each of the primary outcomes. When ORs were not reported in the included study, we manually calculated the OR and its corresponding CI to pool the effect size. Relative risk and hazard ratio were reported in studies summarized in the narrative synthesis if only one study reported a specific factor. For continuous variables, such as age and CD4 count in HIV-infected individuals, we re-categorized these variables as dichotomous and calculated the OR. The re-categorization of age and CD4 count into dichotomous variables was done using study-level data only, with the definitions of younger and older age being study-specific. A forest plot presented the pooled OR for each risk factors for respiratory, hepatic, and hearing sequelae. The heterogeneity of the included studies was assessed using the I^2^ test, and sub-group analysis was conducted to identify the source of heterogeneity by Galbraith radial plot. Publication bias was examined by visually examining the funnel plots and statistically by Eager’s regression statistical test for each significant variable for respiratory, hepatic, and hearing sequelae.

### Ethics approval

Ethical approval was not required as we used publicly available published data.

### Role of funders

KAA is funded by an Australian National Health and Medical Research Council Investigator Grant (APP1196549). This study was also funded by Healy Medical Research Raine Foundation. TYA also received a Higher Degree Research (HDR) Scholarship from Curtin University. The funders had no role in the conception, data collection, analysis, and interpretation of the data, manuscript review, approval of the manuscript, and decision to submit the manuscript for publication.

## Results

**Fig. 1:**
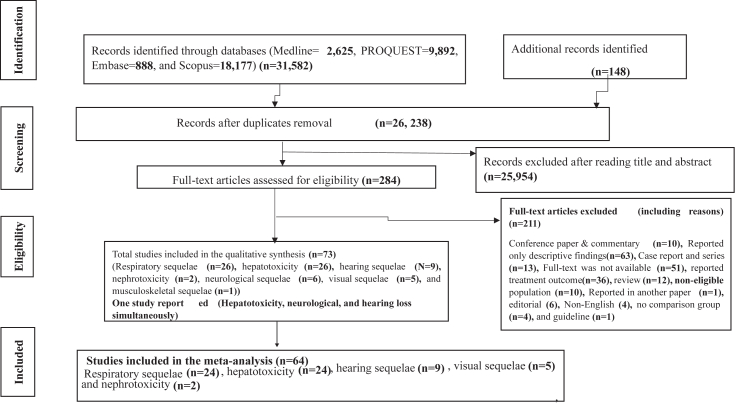
PRISMA flow diagram shows the screening strategy and screening of eligible studies at different levels of the review process.

In this systematic review and meta-analysis, 31,730 articles were identified from database and hand searches and exported to the Endnote reference manager. Of these, 26,238 articles remained after duplicates were removed. Following title and abstract screening, 25,954 articles were excluded based on our inclusion and exclusion criteria, and 284 were screened in a full-text review. A total of 73 articles from 28 countries representing 31,553 TB-treated patients were included in the narrative synthesis (23, 24, 25, 26, 27, 28, 29, 30, 31, 32, 33, 34, 35, 36, 37, 38, 39, 40, 41, 42, 43, 44, 45, 46, 47, 48, 49, 50^,^^51^, ^52^, ^53^, ^54^, ^55^, ^56^, ^57^, ^58^, ^59^, ^60^, ^61^, ^62^, ^63^, ^64^, ^65^, ^66^, ^67^, ^68^, ^69^, ^70^, ^71^, ^72^, ^73^, ^74^, ^75^^,^^76^, ^77^, ^78^, ^79^, ^80^, ^81^, ^82^, ^83^, ^84^, ^85^, ^86^, ^87^, ^88^, ^89^, ^90^, ^91^, ^92^, ^93^, ^94^, ^95^). Of these, 64 studies were included in the meta-analysis (Fig. 1). For records excluded after reading the title and abstract, the reason for exclusion is summarized in a Supplementary File Table S2.

### Characteristics of the included studies

Table 1 provides detailed characteristics of the included studies. The studies were conducted between 1996 and 2023 in 28 different countries, with the greatest number of studies conducted in India (n = 16) South Korea (n = 9), and China (n = 5). The majority (n = 49) of the studies were conducted among an adult population, and only two studies were conducted in children. Most studies (n = 59) were conducted among DS-TB patients, with some (n = 8) conducted among DR-TB patients. The prevalence of DM among TB patients was reported in 14 studies, ranging from 3.57% in China to 27% in South Korea. Nearly half (n = 39) of the studies reported HIV status. Of these 73 studies reviewed, two studies focused solely on HIV-negative patients, 37 studies included both HIV-positive and HIV-negative patients and six studies exclusively dealt with HIV-positive patients. The remaining 28 (38.36%) did not report HIV status. The prevalence of HIV varied from 0.06% to 100% in the six studies that included TB-HIV patients.

**Table 1:** Characteristics of included studies.

First author, year of publication	Year of data collection	Country	Country income	Study design	Study population	Type of TB	Site of TB	Mean/median age	Proportion of male	Sample size	Timing of sequelae
**Lung sequelae**											
Aggarwal D, 2017	2016	India	LMICs	Case-control	Adults	DS-TB	Pulmonary	60.2	89.2	74	Post-treatment
Auld SC, 2021	NR	South Africa	UMICs	Prospective cohort	Adults	TB-HIV	Pulmonary	36	57	134	During treatment
Bajpai J, 2013	NR	India	LMICs	Prospective cohort	Adults	DS-TB	Pulmonary	36	46.21	132	Post-treatment
Chung K, 2011	2000–2008	Taiwan	HICs	Retrospective cohort	NR	DS-TB	Pulmonary	59.3	76.87	134	Post-treatment
Chushkin MI, 2017	2005–2013	Russia	UMICs	Prospective cohort	Adults	DS-TB	Pulmonary	51.1	61.7	214	During treatment
Candela E, 2003	1986–2000	Spain	HICs	Retrospective cohort	both	DS-TB	Pulmonary	28	56.79	81	During treatment
Gupte N, 2019	2016–2018	India	LMICs	Prospective cohort study	adults	DS-TB	Pulmonary	32	52	172	Post-treatment
He M, 2023	2019–2022	China	UMICs	Retrospective cohort	Adults	DS-TB	Pulmonary	65	78.31	249	During treatment
Hoyt KJ, 2019	2015–2017	India	LMICs	Retrospective cohort	Adults	DS-TB	Pulmonary	45	76	173	During treatment
Jung JW, 2015	2008–2012	South Korea	HICs	Cross-sectional	Adults	DS-TB	Pulmonary	59.5	53.3	822	During treatment
Jo YS, 2017	2010–2015	South Korea	HICs	Retrospective cohort	Adults	DS-TB	Pulmonary	63.47	67	195	Post-treatment
Khosa C, 2020	2014–2016	Mozambique	LICs	Prospective cohort	Adults	DS-TB	Pulmonary	29.5	67.74	62	During treatment
Lee SW, 2010	2001	South Korea	HICs	Retrospective cohort	Adults	DS-TB	Pulmonary	53.3	45.9	3687	During treatment
Lisha P, 2012	2008–2010	India	LMICs	Cross-sectional	NR	DS-TB	Pulmonary	47	81	224	Post-treatment
Manji M, 2016	2014	Tanzania	LMICs	Cross-sectional	NR	DS-TB	Pulmonary	NR	60.5	501	During treatment
Mbatchou B, 2016	2008–2012	Cameron	LMICs	Cross-sectional	Adults	DS-TB	Pulmonary	33	54.3	269	Post-treatment
Mpagama S, 2021	NR	Tanzania	LMICs	Cross-sectional	Adults	DS-TB	Pulmonary	45	88	219	Post-treatment
Namusobya M, 2023	2022	Uganda	LICs	Cross-sectional	Adults	Both	Pulmonary	36	55.8	326	Post-treatment
Nihues S, 2015	2013–2014	Brazil	UMICs	Cross-sectional	Adults	DS-TB	Pulmonary	NR	44.63	121	Post-treatment
Nkerreuwem S., 2022	2014–2019	Gambia	LICs	Cross-sectional	Children	DS-TB	Pulmonary	6.5	52.9	159	Post-treatment
Nuwagira E, 2020	2018	Uganda	LICs	Cross-sectional	Adults	MDR-TB	Pulmonary	39	60	95	Post-treatment
Park J, 2023	2008–2009	South Korea	HICs	Cross-sectional	Adults	DS-TB	Pulmonary	55.6	30.13	4911	During treatment
Pasipanodya JG, 2007	2005–2006	USA	HICs	Case-control	Adults	DS-TB	Pulmonary	NR	58.36	317	Post-treatment
Powers M, 2019	1989–1995	USA	HICs	Prospective cohort	Adults	DS-TB	Pulmonary	NR	39.1	2463	During treatment
Radovic M, 2016	2005–2012	Serbia	UMICs	Case-control	Adults	DS-TB	Pulmonary	NR	80	40	During treatment
Soemarwoto R., 2021	2017	Indonesia	LMICs	Case-control	Both	DS-TB	Pulmonary	55.6	NR	64	During treatment
**Hepatic sequelae**											
Ali H, 2013	2008–2011	Ethiopia	LICs	Case-control	NR	TB-HIV	Both	32.6	53	296	During treatment
Anand CAC, 2006	2000–2002	India	LMICs	Case-control	Adults	DS-TB	Both	39.7	52.2	236	During treatment
Araujo-Mariz C, 2016	2006–2012	Brazil	UMICs	Prospective cohort	Adults	TB-HIV	NR	NR	67.1	173	During treatment
Chang KC, 2007	2001	Hong Kong	HICs	Case-control	NR	DS-TB	Both	NR	67.7	288	During treatment
Cusack RP, 2017	2009–2014	Ireland	HICs	Retrospective cohort	Adults	DS-TB	Both	NR	62	275	During treatment
Lima MD, 2012	2004–2007	Brazil	UMICs	Case-control	Adults	TB-HIV	Both	NR	63.46	156	During treatment
Pande JN, 1996	1991–1994	India	LMICs	Case-control	NR	DS-TB	Pulmonary	NR	55.69	492	During treatment
Ergan B, 2017	1997–2007	Turkey	HICs	Retrospective cohort	both	DS-TB	Both	48	49.4	64	During treatment
Jiang F, 2021	2016–2017	China	UMICs	Prospective cohort	Adults	DS-TB	Both	37	64	3325	During treatment
Kar P, 2018	NR	India	LMICs	Case-control	NR	DS-TB	Pulmonary	NR	56	100	During treatment
Kwon YS, 2007	1994–2005	South Korea	HICs	Retrospective cohort	Adults	DS-TB	Both	56.4	61	54	During treatment
Lim J, 2023	2019–2021	South Korea	HICs	Prospective	Adults	DS-TB	Pulmonary	59.9	63	684	During treatment
Makhlouf H, 2008	2004–2005	Egypt	LMICs	Prospective cohort	NR	DS-TB	Both	33.6	44	100	During treatment
Mankhatitham W, 2011	2006–2007	Thailand	UMICs	Retrospective cohort	NR	TB-HIV	NR	36.8	67.2	134	During treatment
Marzuki OA, 2008	2003–2008	Malaysia	UNICs	Case-control	Adults	DS-TB	Both	NR	64.67	184	During treatment
Molla Y, 2021	2017–2019	Ethiopia	LICs	Cross-sectional	Both	DS-TB	Both	NR	45.4	216	During treatment
Raj Mani, S, 2021	2013–2014	India	LMICs	Prospective cohort	Adults	DS-TB	Both	41	61	393	During treatment
Saha A, 2016	2008–2012	India	LMICs	Retrospective cohort	Both	DS-TB	Pulmonary	NR	NR	253	During treatment
Schultz V, 2014	2000–2014	Brazil	UMICs	Retrospective cohort	Adults	DS-TB	Both	48.3	60.87	69	During treatment
Shu CC, 2013	2005–2009	Taiwan	HICs	Retrospective cohort	Adults	DS-TB	Pulmonary	62.2	67.82	926	During treatment
Singla R, 2010	2004–2009	India	LMICs	Case-control	Adults	DS-TB	Both	NR	NR	603	During treatment
Wang J, 2011	2007–2008	Taiwan	HICs	Prospective cohort	Adults	DS-TB	Both	57.6	62.6	360	During treatment
Wang S, 2018	2010–2016	China	UMICs	Retrospective cohort	Adults	DS-TB	Both	47.96	67.1	155	During treatment
Yimer G, 2008	2004–2005	Ethiopia	LICs	Cross-sectional	Adults	DS-TB	Both	26	53.3	197	During treatment
Zeleke A, 2020	2015–2018	Ethiopia	LICs	Cross-sectional	Adults	TB-HIV	Both	41	52.4	84	During treatment
Zhong T, 2021	2014–2019	China	UMICs	Retrospective cohort	Adults	DS-TB	Pulmonary	NR	65.26	757	During treatment
**Hearing sequelae**											
Aznar ML, 2019	2013–2015	Angola	LMICs	Prospective cohort	Both	MDR-TB	Pulmonary	30	57.4	216	During treatment
Chang, K.C, 2008	2001	Hong Kong	HI	Case-control	NR	DS-TB	Both	51.4	78.7	188	During treatment
Harris T, 2012	NR	South Africa	UMICs	Prospective cohort	NR	MDR-TB	NR	36	34	151	During treatment
Hong H, 2020	2014–2017	South Africa	UMICs	Prospective cohort	Both	MDR-TB	NR	35	54	936	During treatment
Merkler AE, 2017	2006–2012	USA	HICs	Retrospective cohort	Adults	DS-TB	Extrapulmonary	50.7	62.99	808	Post-treatment
Sagwa EL, 2015	2004–2014	Nambia	UMICs	Retrospective cohort	NR	MDR-TB	NR	35.69	56.09	353	During treatment
Seddon J, 2012	2009–2010	South Africa	UMICs	Retrospective cohort	children	MDR-TB	Both	3.6	21.51	94	During treatment
Sharma, 2016	2012–2014	India	LMICs	Retrospective cohort	Adults	MDR-TB	NR	37.6	66.67	100	During treatment
Sogebi O, 2017	2015	Nigeria	LMICs	Prospective cohort	Adults	DS-TB	NR	34.6	62.9	70	During treatment
**Neurological sequelae**											
Kalita J, 2007	NR	India	LMICs	Prospective cohort	Both	DS-TB	Extrapulmonary	33.2	59.58	65	During treatment
Mittal S, 2021	2016–2019	India	LMICs	Case-control	Both	DS-TB	Extrapulmonary	38.1	38.1	105	During treatment
Popoca-Rodriguze, 2021	2010–2019	Mexico	UMICs	Cross-sectional	Adults	DS-TB	Extrapulmonary	NR	69.23	104	During treatment
Tanaviriyachai T, 2023	2016–2021	Thailand	UMICs	Retrospective cohort	Adults	DS-TB	NR	57.2	49.57	115	During treatment
Zhao J, 2022	2013–2020	China	UMICs	Retrospective cohort	Adults	DS-TB	Extrapulmonary	45.9	67	78	During treatment
**Visual sequelae**											
Chen H, 2012	2000–2008	Taiwan	HICs	Case-control	Adults	DS-TB	NR	NR	66.15	1152	During treatment
Jin KW, 2018	2014–2016	South Korea	HICs	Retrospective cohort	Adults	DS-TB	Both	45.5	47.1	84	During treatment
Merkler A., 2017	2006–2013	USA	HICs	Retrospective cohort	Adults	DS-TB	Extrapulmonary	50.7	62.99	808	Post-treatment
Sinha, 2009	2008–2009	India	LMICs	Prospective cohort	Adults	DS-TB	Pulmonary	30	58.4	101	During treatment
Verma R, 2019	2015–2017	India	LMICs	Prospective cohort	NR	DS-TB	Both	NR	46.53	101	During treatment
**Nephrotic sequelae**											
Kim EJ, 2018	2005–2016	South Korea	HICs	Case-control	Adults	DS-TB	Extrapulmonary	52.8	46.43	56	During treatment
Perumal, 2018	2011–2013	South Africa	UMICs	Retrospective cohort	Adults	MDR-TB	NR	33	46.6	215	During treatment
**MSD**											
Ha YJ, 2019	2004–2016	South Korea	HICs	Case-control	Adults	DS-TB	NR	67.7	79.6	49	During treatment

**Both:** Children and adults, **DS-TB:** Drug-susceptible tuberculosis, **HICs:** High-Income countries, **LICs:** Low-Income countries, **LMICs:** Lower-Middle Income Countries, **MDR-TB:** Multi-drug-resistant Tuberculosis, **MSD:** musculoskeletal Disorder, **NR:** Not Reported, **TB/HIV:** Tuberculosis and HIV co-infection, **UMICs:** Upper-Middle-Income Countries, and **USA:** United States of America.

We reported the mean age for studies that presented the mean age of study participants and the median age for studies that presented the median age.

**Timing of sequelae:** The term “post-treatment” and “during treatment” refer to the time point of sequelae assessment. “During treatment” means the sequelae were assessed while the patient was still undergoing TB treatment, whereas “Post-treatment” means the assessment was done after completing TB treatment.

### Risk factors associated with post-TB lung sequelae

A total of 26 studies reported on risk factors associated with post-TB lung sequelae assessed between 6 months and two years after the completion of TB treatment. These risk factors included socio-demographic, behavioral, treatment, co-morbidity, radiological, environmental, and clinical factors (Supplementary File: Table S3). We conducted a meta-analysis of 24 studies that reported the association of risk factors with lung sequelae.

**Fig. 2:**
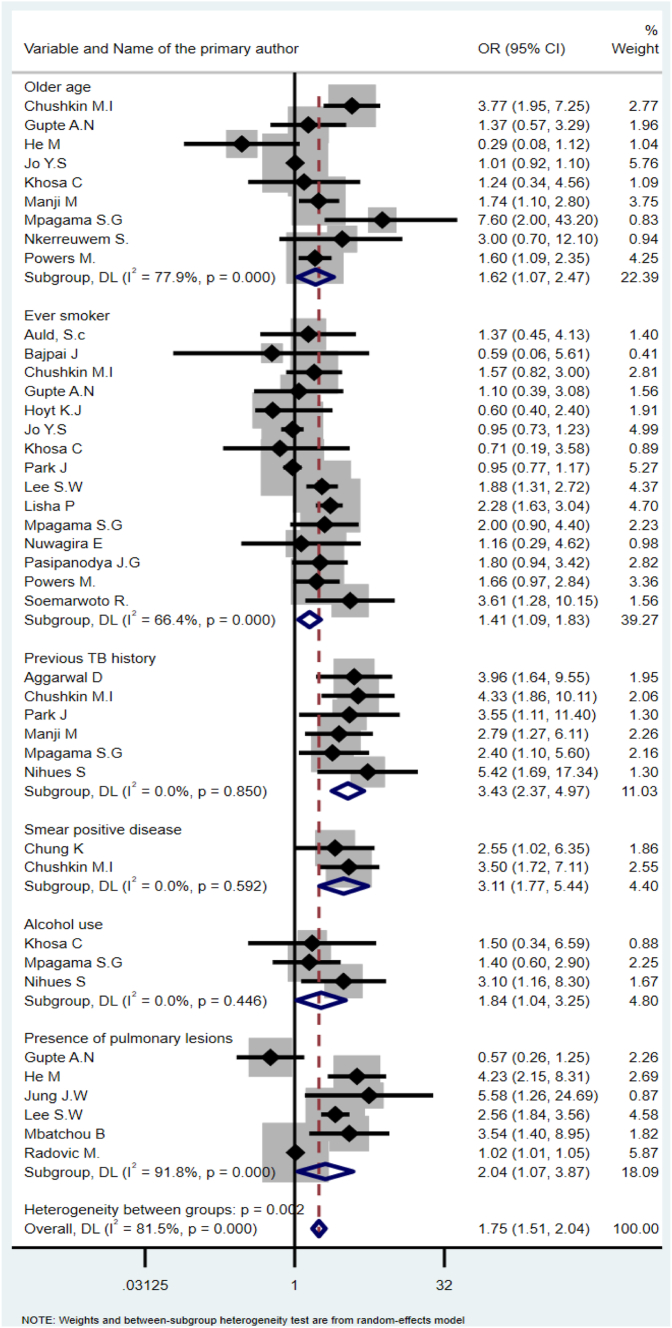
Forest plot summarizing risk factors for lung sequelae.

The pooled effect from nine studies that reported on age showed that older age increased the odds of lung sequelae by 62% (OR = 1.62, 95% CI:1.07–2.47). The pooled results from six studies that reported the effect of previous TB treatment found that a history of previous TB treatment increased the risk of lung sequelae more than threefold (OR = 3.43, 95% CI: 2.37–4.97). The pooled effect from 15 studies that evaluated smoking found 41% higher odds of lung sequelae among smokers compared to non-smokers (OR = 1.41, 95% CI: 1.09–1.83). Alcohol intake also significantly increased the odds of lung sequelae (OR = 1.84, 95% CI: 1.04–3.25). The pooled results from two studies showed that smear-positive patients had nearly three times higher risk of developing lung sequelae compared to those with a smear-negative result (OR = 3.11, 95% CI:1.77–6.44). The pooled results from six studies showed that patients with radiographic pulmonary lesions at baseline had higher odds of lung sequelae compared to TB survivors without pulmonary lesions (OR = 2.04, 95% CI: 1.07–3.87) (Fig. 2).

### Risk factors associated with post-TB liver injury

**Fig. 3:**
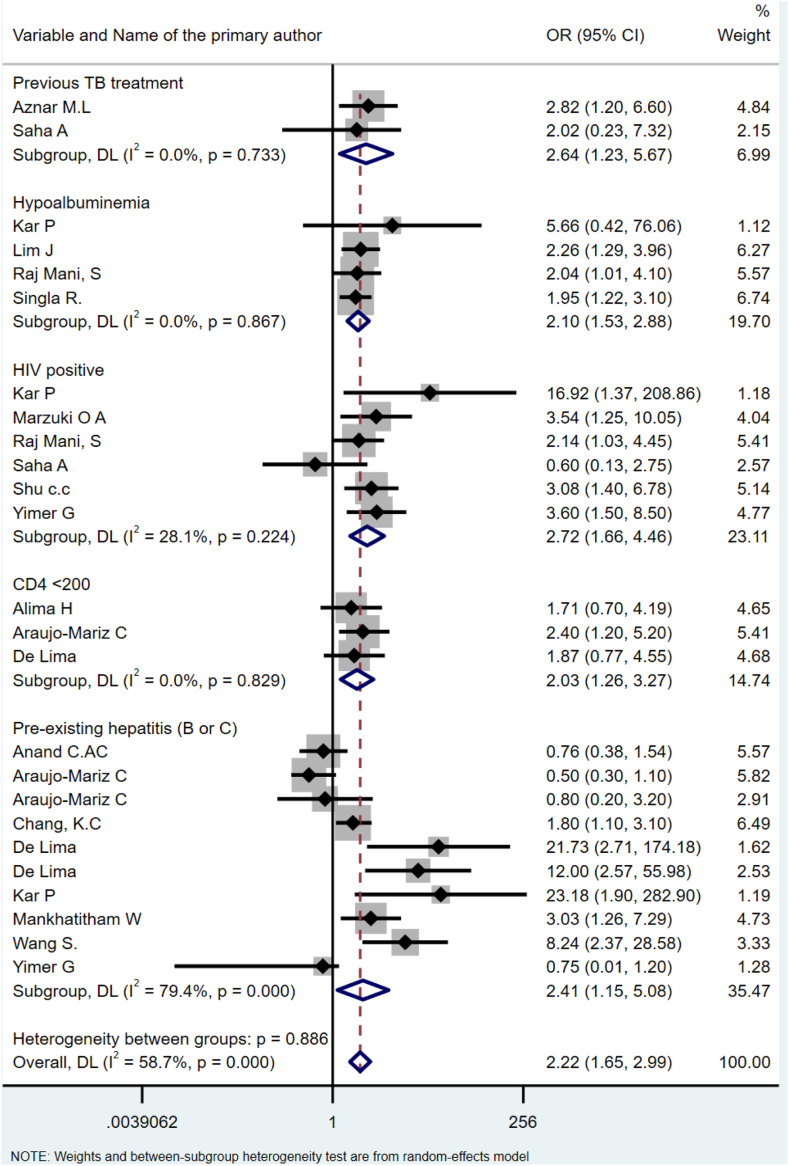
Forest plot for significant risk factors for acute liver injury.

A total of 26 studies reported on risk factors associated with post-TB liver injury (Supplementary File S3: Table S4), with 24 studies included in the meta-analysis. The pooled effect of three studies showed that a CD4 cell count <200 mm^3^ in HIV-infected individuals was positively associated with liver injury compared to patients with a CD4 cell count ≥200 mm^3^ (OR = 2.03, 95%CI: 1.26–3.27). Pre-existing hepatitis (serology)was reported in 10 studies, and the pooled effect showed that the odds of hepatotoxicity were nearly 2.5 times higher compared to patients with no history of hepatitis (OR = 2.41, 95%CI: 1.16–6.08). The pooled effect of previous TB treatments in two studies revealed higher odds of liver injury than newly treated TB patients (OR = 2.64, 95%CI: 1.22–6.67). Hypoalbuminemia, pooled from four studies, increased the odds of developing liver injury when compared to patients with normal levels of blood albumin (OR = 2.10, 95%CI: 1.53–2.88). HIV co-infection, reported in six studies, showed a positive association with liver injury (OR = 2.72,95%CI: 1.66–4.46) (Fig. 3).

### Risk factors associated with post-TB hearing sequelae

**Fig. 4:**
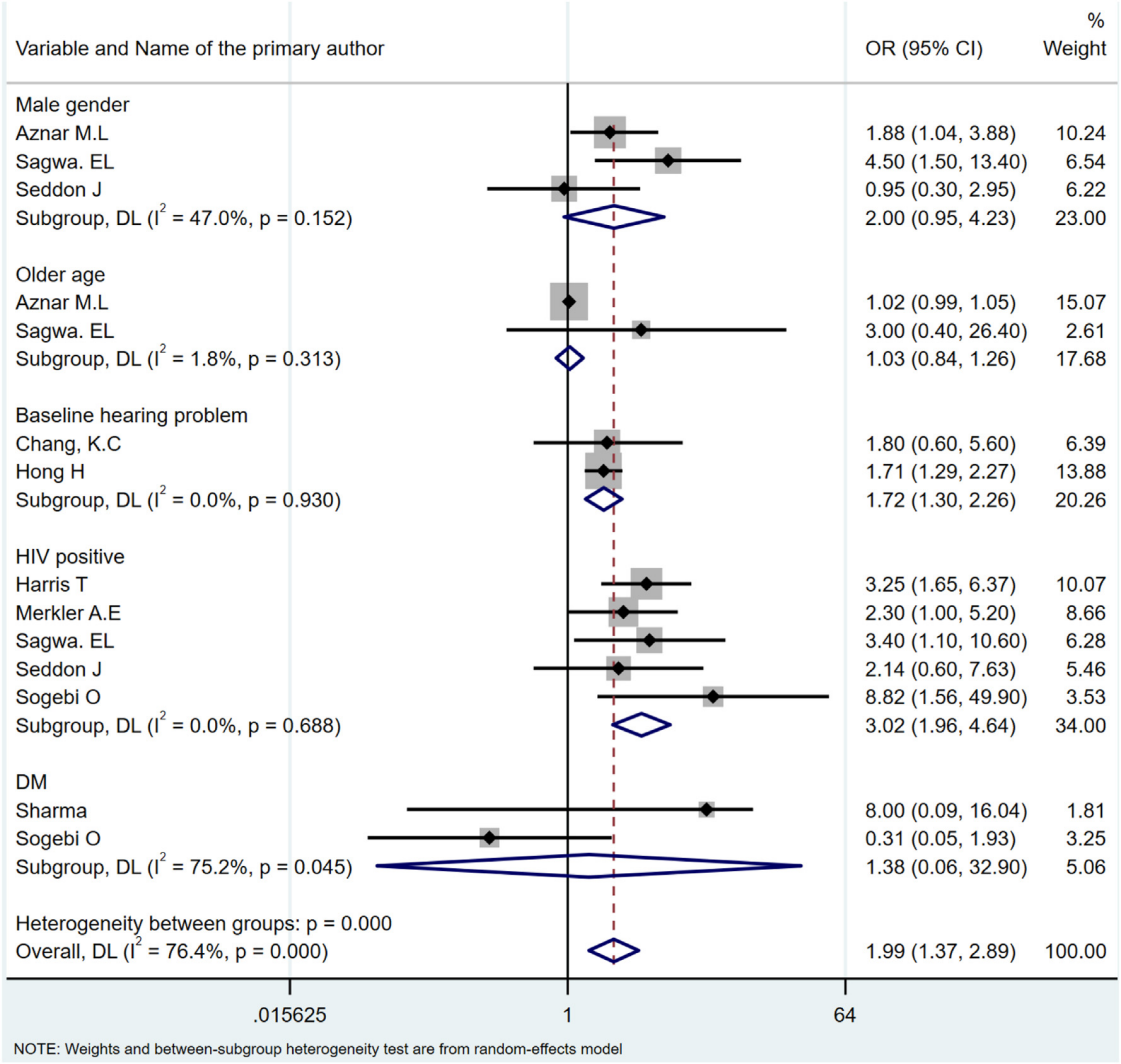
Forrest plot showing the risk factors for hearing sequelae.

Nine studies evaluated the risk factors associated with post-TB hearing sequelae (Supplementary File: Table S5). These studies showed that baseline hearing problems (OR = 1.72, 95% CI: 1.30, 2.26) and HIV co-infection (OR = 3.02, 95%CI: 1.96–4.64) were positively associated with post-TB hearing loss. Findings from single studies showed that pre-existing hepatitis,^31^ previous TB treatment history,^28^ longer duration of treatment (>20 months),^28^ being on pyrazinamide-containing regimens,^31^ male gender,^78^ and weekly aminoglycoside treatment^43^ were positively associated with hearing loss. Conversely, low BMI at admission was negatively associated with hearing loss (Fig. 4).

### Risk factors associated with post-TB neurological sequelae

Six studies investigated the risk factors associated with neurological sequelae. Due to the lack of an adequate number of studies, a meta-analysis was not conducted. However, single studies showed that BMI >25, kyphosis angle >30, older age, cord signal change, longer duration of treatment, canal encroachment >50%, and focal motor deficit were positively associated with post-TB neurological sequelae. In contrast, a good level of consciousness was negatively associated with neurological sequelae in patients treated for TB^49^ (Supplementary File: Table S6).

### Risk factors associated with post-TB visual sequelae

**Fig. 5:**
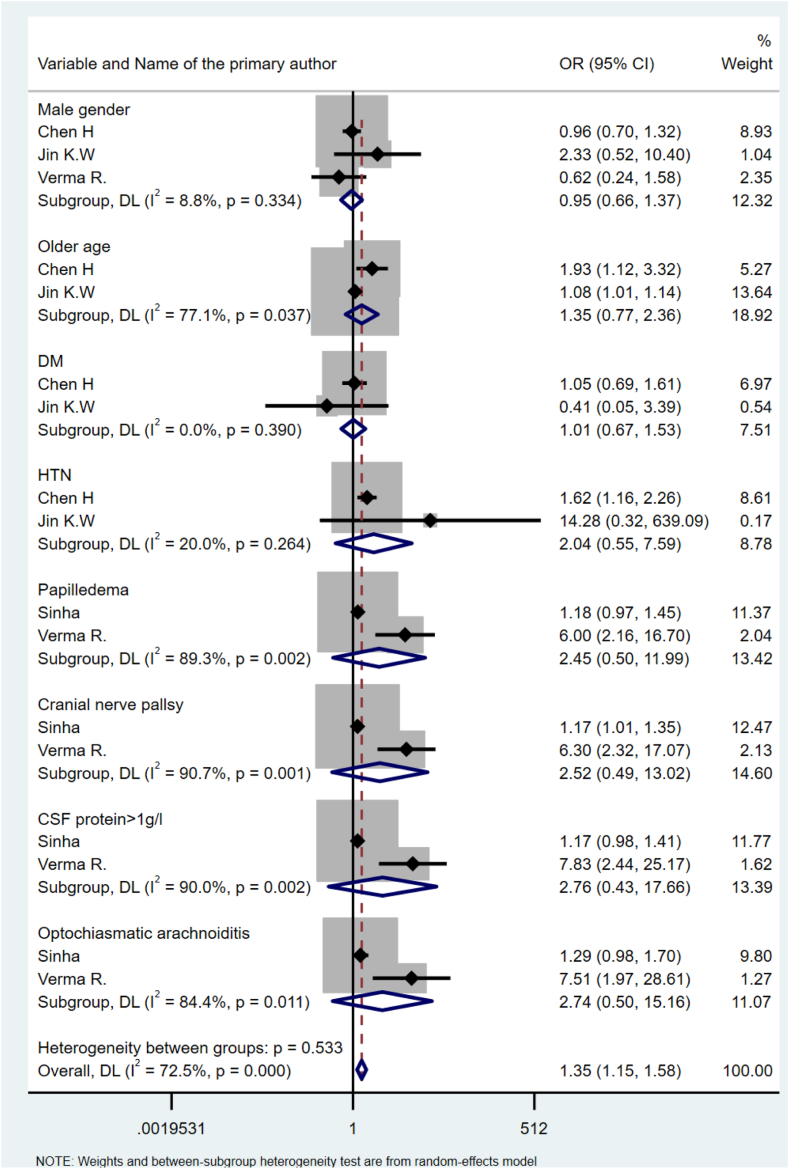
Forest plot summarizing risk factors for visual sequelae.

A range of socio-demographic, TB treatment characteristics, and central nervous system (CNS)-related risk factors were investigated for visual sequelae. A meta-analysis was conducted, revealing that none of the included variables were statistically significant individually (except for optochiasmatatic arachnoiditis), but the overall effect showed statistical significance (Fig. 5). However, our narrative synthesis identified several risk factors associated with visual sequelae, including older age,^33^^,^^47^ renal disease,^33^ hypertension,^33^ longer treatment duration,^47^ miliary TB,^89^ cranial nerve palsy,^89^ cerebrospinal fluid (CSF) protein > 1gr/dl,^85^ altered sensorium,^89^ and papilledema.^89^ Inconsistent findings were reported on the effect of hypertension,^47^ CSF protein >1gr/dl,^89^ and papilledema^85^ on visual sequelae in patients treated for TB (Supplementary File: Table S7).

### Risk factors associated with post-TB renal and musculoskeletal sequelae

**Fig. 6:**
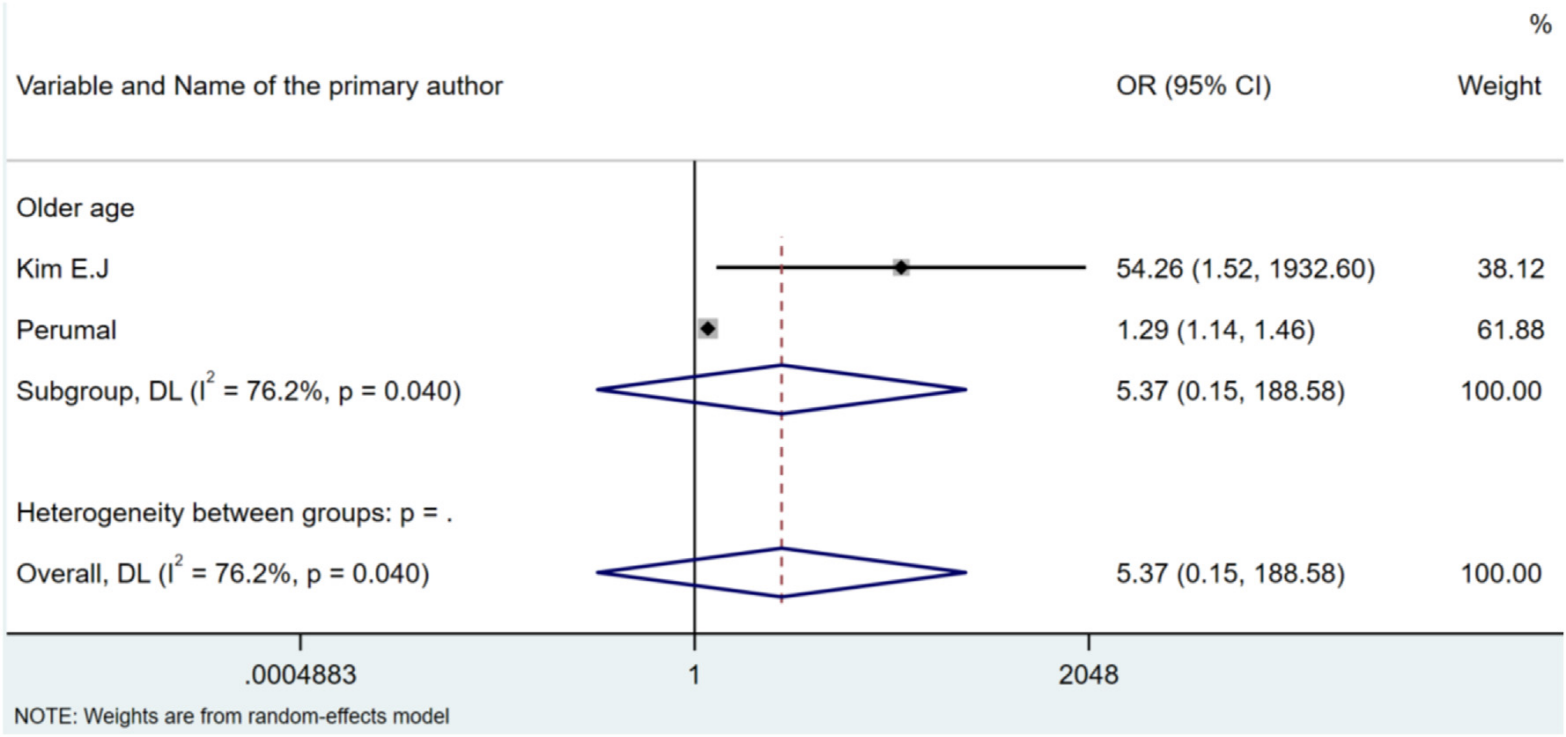
Forest plot summarizing risk factors for renal impairment.

Two studies that reported risk factors for post-TB renal sequelae showed that older age, kanamycin-based regimens, and acute renal failure were positively associated with post-TB renal sequelae in TB survivors. However, the meta-analysis found that older age was not significantly associated with renal impairment (OR = 5.37, 95% CI: 0.15–188.59) (Fig. 6). Only a single study investigated risk factors associated with musculoskeletal sequelae, showing that BMI >18.5, chronic kidney disease, and pre-treatment hyperuricemia^40^ were positively associated with musculoskeletal sequelae (Supplementary File: Table S8).

### Quality assessment

The overall quality assessment of the included studies ranged from poor to high, with a median score of 7 points and an Inter Quartile Range (IQR) of 2 points. Out of 73 included studies, the majority (73.6%) had five to seven points, regarded as moderate-quality studies (Supplementary File: Table S9).

### Publication bias and heterogeneity assessment

Funnel plots and the Egger regression findings revealed no publication bias in the significant risk factors identified for respiratory, hepatic, and hearing sequelae (Supplementary File Figs S4, S7–S19). Similarly, the Galbraith plots for all significant risk factors revealed that there was no significant heterogeneity (Supplementary File Figs. S20–S32).

## Discussion

Post-TB sequelae present a significant challenge in the management of TB survivors, often leading to persistent health issues even after successful treatment. This systematic review and meta-analysis aimed to identify risk factors associated with long-term physical sequelae among TB survivors. Our findings, based on a comprehensive synthesis of 73 articles from 28 countries, representing 31,553 TB-treated patients, identified sociodemographic and clinical factors contributing to the development of different types of post-TB sequelae.

Risk factors associated with post-TB lung sequelae include older age, previous TB treatment, smoking, alcohol use, smear positivity at baseline, and pulmonary lesions on radiological examinations at baseline. Despite TB being more common among younger populations, our findings showed that the elderly population is at higher risk of developing post-TB lung sequelae, potentially due to age-related immune system changes and treatment-related challenges.^96^^,^^97^ Smoking and alcohol consumption were identified as significant factors for lung sequelae. This is because smoking causes delayed culture conversion,^98^ and poor treatment outcomes (loss to follow-up and treatment failure) in TB survivors^99^ that further leads to long-term respiratory residuals. The higher burden among alcohol users could be due to the reduction of host defenses of airways,^100^ the dysfunction of the alveolar epithelial barrier or macrophages^101^), and severe depletion of glutathione stores within the alveolar space^102^ in alcoholic people.

Similarly, prior TB treatment and the presence of pulmonary lesions further increase the risk of post-TB lung sequelae. Previous TB treatment causes long-term respiratory sequelae as a result of structural damage, metabolic derangement, and infectious complications.^103^ This finding is in line with another systematic review that found structural lung abnormalities such as cavitations, fibrosis, and bronchiectasis, leading to abnormal airway physiology and lung function.^104^ These findings indicate the importance of targeted interventions for high-risk groups, particularly considering the aging global demographic and increasing TB survivor population.^105^^,^^106^ Integrating multidisciplinary approaches into rehabilitation and after-care programs could help address the challenges faced by these populations, while interventions targeting smoking and alcohol abuse should be integrated into post-TB health programs to mitigate associated risks effectively.^107^, ^108^, ^109^

Our study identified several risk factors associated with an increased risk of liver injury following TB treatment, including a CD4 count less than 200 mm^3^ in HIV-infected individuals, pre-existing hepatitis, previous TB treatment history, hypo-albuminemia, and HIV co-infection. These findings showed the intricate interplay between immunosuppression, pre-existing conditions, and treatment-related factors in the development of hepatic sequelae. HIV-coinfected individuals may require taking multiple medications for both diseases, which can potentially lead to impaired liver function.^110^, ^111^, ^112^

Previous studies also showed that chronic hepatitis increases the risk of hepatotoxicity among TB survivors^113^ through replication of the virus that further leads to liver injury.^114^^,^^115^ Moreover, the potential liver dysfunction that happens due to chronic hepatitis, could impair the disposition of anti-TB drugs, which increases the accumulation of more toxic metabolites.^116^ Hypoalbuminemia also leads to hepatotoxicity among TB patients due to triggering inflammatory responses such as tumor necrosis factor (TNF) and interleukin (IL)-6 in TB patients, which leads to increased vascular permeability, increasing degradation, and decreasing synthesis of albumin.^117^ Hypoalbuminemia significantly affects the transport of drugs and causes increased antibiotic clearance.^118^ Anti-TB drugs such as isoniazid, rifampicin, and pyrazinamide are the main causes of hepatotoxicity in TB survivors, with the majority of hepatotoxicity cases occurring during the first weeks of the intensive phase of anti-TB treatment.^119^ Clinicians need to be vigilant in monitoring liver function among individuals with these risk factors, and strategies should be devised to minimize hepatic complications during and after TB treatment.

Baseline hearing problems and HIV co-infection were identified as significant risk factors for post-TB hearing loss. These findings showed the need for routine hearing assessments and specialized care for individuals with TB and HIV co-infection. Several studies have reported that second-line TB medications such as Kanamycin, Amikacin, and Capreomycin can increase the risk of hearing loss in TB patients with pre-existing hearing problem.^120^, ^121^, ^122^ However, in our study, most of the included studies were conducted in DS-TB cases and only a single study reported that aminoglycoside agents were associated with hearing loss.

Our systematic review and meta-analysis provide a comprehensive understanding of the socio-demographic, behavioral, and clinical factors influencing long-term physical post-TB sequelae. However, several limitations need to be considered when interpreting the findings. Potentially important variables such as pre-existing respiratory problems (e.g., bronchial asthma and COPD), poor functional status at TB diagnosis, unemployment, poor social support, and mental health status (i.e., depression, anxiety, and stress) are lacking in the existing literature and not included in our systematic review. Thus, more primary studies are needed to incorporate missed variables and perform risk prediction modeling to identify high-risk groups for targeted interventions and preventive measures. This systematic review was also limited to articles published in English. The lack of data restricted us from conducting a meta-analysis for some important post-TB sequelae, including neurological and musculoskeletal outcomes. The low number of studies in neurological and musculoskeletal sequelae indicates significant knowledge gaps in the existing evidence on risk factors for neurological and musculoskeletal post-TB sequelae, indicating the need for further studies. The inability to obtain full texts for 51 articles may affect the robustness of the findings. Another limitation is the potential for type II error due to small sample sizes in some studies, meaning some important predictors of post-TB sequelae might not have been detected. Future studies with larger sample sizes are needed to identify potential risk factors. The quality of evidence could be affected by the significant number of cross-sectional and retrospective studies included in the systematic review. TB sequelae research is dynamic, and there could be misclassification bias due to the variation used to ascertain the outcome of interest. We also acknowledged the challenge of defining a precise temporal boundary between the end of treatment and assessing post-TB sequelae. We could not identify risk factors for DS-TB and DR-TB separately for respiratory, acute liver injury, visual, and renal sequelae due to the limited number of DR-TB studies.

Our systematic review and meta-analysis identified significant risk factors for post-TB sequelae in TB survivors, including older age, history of TB treatment, smoking, alcohol use, baseline smear positivity, and pulmonary lesions. Post-TB treatment follow-up and programmatic support aimed at high-risk individuals are important for mitigating the growing burden of post-TB sequelae.

## Contributors

**Conceptualization:** Temesgen Yihunie Akalu, Kefyalew Addis Alene, Archie C. A. Clements, Megan B. Murray.

**Data curation:** Temesgen Yihunie Akalu, Archie C. A., Megan B. Murray, Alemneh Mekuriaw Liyew, Beth Gilmour, Kefyalew Addis Alene.

**Formal analysis:** Temesgen Yihunie Akalu.

**Investigation:** Temesgen Yihunie Akalu, Megan B. Murray, Alemneh Mekuriaw Liyew, Beth Gilmour, Archie C. A., Kefyalew Addis Alene.

**Methodology:** Temesgen Yihunie Akalu & Kefyalew Addis Alene.

**Supervision:** Archie C. A. Clements, Kefyalew Addis Alene.

**Validation:** Temesgen Yihunie Akalu, Archie C. A. Clements, Kefyalew Addis Alene.

**Writing—original draft:** Temesgen Yihunie Akalu.

**Writing—review & editing:** Archie C. A. Clements, Megan B. Murray, Alemneh Mekuriaw Liyew, Beth Gilmour, Kefyalew Addis Alene.

All authors had full access to the data and had final responsibility for the decision to submit for publication. Temesgen Yihunie Akalu and Kefyalew Addis Alene accessed and verified the data.

## Data sharing statement

Data will be available upon request from the corresponding author.

## Declaration of interests

All authors declare that they have no conflict of interest.

## Appendix ASupplementary data

Supplementary Figs. S1–S32 and Tables S1–S8

Translated abstract
