# Supplementary material for: Risk factors associated with post-tuberculosis sequelae: a systematic review and meta-analysis
Source: eClinicalMedicine. 2024 Oct 21;77:102898. doi: 10.1016/j.eclinm.2024.102898 (PMC11535315; doi:10.1016/j.eclinm.2024.102898)
Supplement: Supplementary Figs. S1–S32 and Tables S1–S8 [file mmc1.docx]

**Supplementary files**

# **Supplementary file : Definition of crucial terms**

**Long-term physical sequelae** are defined as Tuberculosis (TB) patients who had neurological function impairment (peripheral neuropathy, neurological deficit, hemiplegia, seizures, palsy, paraplegia, and paralysis), visual impairment (i.e... blindness, color vision impairment), hearing impairment (i.e... deafness or hearing loss), musculoskeletal impairment (spinal deformity, orthopaedic deformity, spondylitis, osteomyelitis, kyphosis, and chronic arthritis), renal impairment (e.g., renal failure and nephrotoxicity), hepatic failure (hepatotoxicity), respiratory impairment (respiratory insufficiency, destroyed lung, lung function impairment, Chronic Obstructive Pulmonary Disease (COPD), bronchiectasis, chronic airway obstruction, and lung fibrosis.

**The risk factor** **of post-TB sequelae** is defined as any variable that increases the likelihood of developing long-term sequelae following TB.

**Radiographical lesions** include fibrosis, cavities, bronchiectasis, calcifications, atelectasis, and nodules.

**Supplementary File: Table 1:** search strategies for the included studies

| # | **12/12/2023 Searching strategy** | **Yields** |
| --- | --- | --- |
| **Medline (Ovid)** | | |
| 1 | ("tuberculosis"[MeSH] OR "mycobacterium tuberculosis"[MeSH] OR "tuberculosis"[Title] OR "tuberculoses"[ Title] | 130,126 |
| 2 | ("lung disease*" or "fibrosis" or "COPD" or "chronic obstructive pulmonary disease" or "bronchiectasis" or "pulmonary atrophy" or "lung atrophy" or "atelectasis" or "pulmonary embolism" or "respiratory function test" or "pulmonary disease" or "scar" or "destroyed lung" or "chronic airway obstruction" or "lung fibrosis") [title] | 130,198 |
| 3 | (("neurologic*" or "visual" or "vision" or "hearing" or "musculoskeletal" or "renal" or "respiratory" or "hepatic" or "cardiac" or "pulmonary") adj3 ("impairment*" or "failure*" or "abnormal*")) [title] | 196,700 |
| 4 | ("peripheral neuropathy" or "neurological deficit" or "hemiplegia" or "seizures" or "palsy" or "paraplegia" or "paralysis" or "neurologic" or "spinal deformity" or "orthopaedic deformity" or "spondylitis" or "osteomyelitis" or "kyphosis" or "chronic arthritis") [title] | 88237 |
| 5 | ("Disabled Persons" or "Hearing Loss" or "deaf*" or "Vision Disorders" or "color vision impairment" or "blind*" or "Heart Failure" or "Chemical and Drug Induced Liver Injury" or "Granuloma" or "Renal Insufficiency" or "Peripheral Nervous System Diseases" or "nephrotoxicity" or "renal toxicity" or "hepatotoxicity") [title] | 166,189 |
| 6 | (sequela* OR complication* OR illness* OR prognos* OR function* OR chronic* OR consequence* OR burden* OR disabl* OR catastroph* or impair*) AND ("long-term" or “longterm”) [title] | 90,102 |
| 7 | “Risk factors” OR “predictor*” OR “determinants” OR “influence” OR “Reasons” OR “Causes” OR “determining factor*” OR “prognosticator” OR “relative risk” OR risk$.ti,ab OR factor$.ti,ab OR odds ratio$.ti,ab OR predict$.ti,ab OR correlate$.ti,ab OR etiol$.ti,ab OR rate$.ti,ab OR associat$.ti,ab OR discriminat$.ti,ab [title] | 8366404 |
| 8 | 2 OR 3 OR 4 OR 5 OR 6 | 646929 |
| 9 | 1 AND 7 AND 8 (limit English language and humans) | 2,625 |
| **Embase** | | |
| 1 | ("tuberculosis"[MeSH] OR "mycobacterium tuberculosis"[MeSH] OR "tuberculosis"[Title/Abstract] OR "tuberculoses"[ Title/Abstract] | 80,737 |
| 2 | ("lung disease*" or "fibrosis" or "COPD" or "chronic obstructive pulmonary disease" or "bronchiectasis" or "pulmonary atrophy" or "lung atrophy" or "atelectasis" or "pulmonary embolism" or "respiratory function test" or "pulmonary disease" or "scar" or "destroyed lung" or "chronic airway obstruction" or "lung fibrosis") | 209,558 |
| 3 | (("neurologic*" or "visual" or "vision" or "hearing" or "musculoskeletal" or "renal" or "respiratory" or "hepatic" or "cardiac" or "pulmonary") adj3 ("impairment*" or "failure*" or "abnormal*")) | 53,524 |
| 4 | ("peripheral neuropathy" or "neurological deficit" or "hemiplegia" or "seizures" or "palsy" or "paraplegia" or "paralysis" or "neurologic" or "spinal deformity" or "orthopaedic deformity" or "spondylitis" or "osteomyelitis" or "kyphosis" or "chronic arthritis") | 108,352 |
| 5 | ("Disabled Persons" or "Hearing Loss" or "deaf*" or "Vision Disorders" or "color vision impairment" or "blind*" or "Heart Failure" or "Chemical and Drug Induced Liver Injury" or "Granuloma" or "Renal Insufficiency" or "Peripheral Nervous System Diseases" or "nephrotoxicity" or "renal toxicity" or "hepatotoxicity") | 237,841 |
| 6 | ((sequela* or complication* or illness* or prognos* or function* or chronic* or consequence* or burden* or disabl* or catastroph* or impair*) and ("long-term" or "longterm")) | 32019 |
| 7 | “Risk factors” OR “predictor*” OR “determinants” OR “influence” OR “Reasons” OR “Causes” OR “determining factor*” OR “prognosticator” OR “relative risk” OR risk$.ti,ab OR factor$.ti,ab OR odds ratio$.ti,ab OR predict$.ti,ab OR correlate$.ti,ab OR etiol$.ti,ab OR rate$.ti,ab OR associat$.ti,ab OR discriminat$.ti,ab | 112,760,019 |
| 8 | 2 or 3 or 4 or 5 or 6 | 631,388 |
| 9 | 1 and 7 and 8 | 888 |
| **PROQUEST** | | |
| 1 | title(("tuberculosis"OR "mycobacterium tuberculosis"[MeSH]) ) | 163,642 |
| 2 | (("lung disease" OR "lung diseases") or "fibrosis" or "COPD" or "chronic obstructive pulmonary disease" or "bronchiectasis" or "pulmonary atrophy" or "lung atrophy" or "atelectasis" or "pulmonary embolism" or "respiratory function test" or "pulmonary disease" or "scar" or "destroyed lung" or "chronic airway obstruction" or "lung fibrosis") | 1,488,453 |
| 3 | (("neurologic*" or "visual" or "vision" or "hearing" or "musculoskeletal" or "renal" or "respiratory" or "hepatic" or "cardiac" or "pulmonary") adj3 ("impairment*" or "failure*" or "abnormal*")). | 453 |
| 4 | ("peripheral neuropathy" or "neurological deficit" or "hemiplegia" or "seizures" or "palsy" or "paraplegia" or "paralysis" or "neurologic" or "spinal deformity" or "orthopaedic deformity" or "spondylitis" or "osteomyelitis" or "kyphosis" or "chronic arthritis") | 1,280,412 |
| 5 | ("Disabled Persons" or "Hearing Loss" or "deaf*" or "Vision Disorders" or "color vision impairment" or "blind*" or "Heart Failure" or "Chemical and Drug Induced Liver Injury" or "Granuloma" or "Renal Insufficiency" or "Peripheral Nervous System Diseases" or "nephrotoxicity" or "renal toxicity" or "hepatotoxicity") | 3,624,261 |
| 6 | ((sequela* or complication* or illness* or prognos* or function* or chronic* or consequence* or burden* or disabl* or catastroph* or impair*) and ("long-term" or "longterm")). | 3,645,269 |
| 7 | “Risk factors” OR “predictor*” OR “determinants” OR “influence” OR “Reasons” OR “Causes” OR “determining factor*” OR “prognosticator” OR “relative risk” OR risk$.ti,ab OR factor$.ti,ab OR odds ratio$.ti,ab OR predict$.ti,ab OR correlate$.ti,ab OR etiol$.ti,ab OR rate$.ti,ab OR associat$.ti,ab OR discriminat$.ti,ab | 17,824,466 |
| 8 | 2 OR 3 OR 4 OR 5 OR 6 | 8,290,599 |
| 9 | 1 AND 7 AND 8 (Limit to English language and articles) | 14,509 |
| 10 | Limit 9 by Limit to ProQuest Central, articles, and full text) | 9,892 |
| **Scopus** | | |
| 1 | ( "tuberculosis" OR "mycobacterium tuberculosis" ) | 120,568 |
| 2 | (("lung disease" OR "lung diseases") or "fibrosis" or "COPD" or "chronic obstructive pulmonary disease" or "bronchiectasis" or "pulmonary atrophy" or "lung atrophy" or "atelectasis" or "pulmonary embolism" or "respiratory function test" or "pulmonary disease" or "scar" or "destroyed lung" or "chronic airway obstruction" or "lung fibrosis") | 427,665 |
| 3 | "neurologic*" or "visual" or "vision" or "hearing" or "musculoskeletal" or "renal" or "respiratory" or "hepatic" or "cardiac" or "pulmonary" OR "impairment*" or "failure*" or "abnormal*" | 3,766,840 |
| 4 | ("peripheral neuropathy" or "neurological deficit" or "hemiplegia" or "seizures" or "palsy" or "paraplegia" or "paralysis" or "neurologic" or "spinal deformity" or "orthopaedic deformity" or "spondylitis" or "osteomyelitis" or "kyphosis" or "chronic arthritis") | 488,804 |
| 5 | ("Disabled Persons" or "Hearing Loss" or "deaf*" or "Vision Disorders" or "color vision impairment" or "blind*" or "Heart Failure" or "Chemical and Drug Induced Liver Injury" or "Granuloma" or "Renal Insufficiency" or "Peripheral Nervous System Diseases" or "nephrotoxicity" or "renal toxicity" or "hepatotoxicity") | 798,353 |
| 6 | ((sequela* or complication* or illness* or prognos* or function* or chronic* or consequence* or burden* or disabl* or catastroph* or impair*) and ("long-term" or "longterm")) | 355,671 |
| 7 | "Risk factors" OR "predictor*" OR "determinants" OR "influence" OR "Reasons" OR "Causes" OR "determining factor*" OR "prognosticator" OR "relative risk" OR "risk$.ti,ab" OR "factor$.ti,ab" OR "odds ratio$.ti,ab" OR "predict$.ti,ab" OR "correlate$.ti,ab" OR "etiol$.ti,ab" OR "rate$.ti,ab" OR "associat$.ti,ab" OR "discriminat$.ti,ab" | 3,121,225 |
| 8 | 2 OR 3 OR 4 OR 5 OR 6 | 4,520,352 |
| 9 | 1 AND 7 AND 8 | 18,177 |

**Supplementary File: Table 2:** Reasons for exclusion of studies

| **S.N** | **First author & year** | **Title** | **Journal** | **Reasons for exclusion** |
| --- | --- | --- | --- | --- |
|  | Ahmed H, 2023 | Pulmonary Hypertension in Patients with Treated Pulmonary Tuberculosis: Analysis of 14 Consecutive Cases | Clinical Medicine Insights. Circulatory, Respiratory and Pulmonary Medicine | Report only descriptive findings |
|  | Appana, D 2016 | An audiological profile of patients infected with multi-drug resistant tuberculosis at a district hospital in KwaZulu-Natal | South African Journal of Communication Disorders | Report only descriptive findings |
|  | Arsalan R 2015 | Isoniazid-induced motor-dominant neuropathy | Journal of the Pakistan Medical Association | Case report |
|  | Atita E 2005 | Adolescent age is an independent risk factor for abnormal spirometry among people living with HIV in Kenya | AIDS | Different population (includes general population) |
|  | Allwood B.W. 2021 | Post-Tuberculosis Lung Disease: Clinical Review of an Under-Recognized Global Challenge | Respiration | Review |
|  | Allwood B.W, 2020 | Transition from Restrictive to Obstructive Lung Function Impairment During Treatment and Follow-Up of Active Tuberculosis | International journal of chronic obstructive pulmonary disease | Report only descriptive findings |
|  | Al-Sayyad M.J, 2011 | Tuberculous arthritis revisited as a forgotten cause of monoarticular arthritis | Annals of Saudi Medicine | Case report and series |
|  | Anonymous 2023 | Chronic Pulmonary Aspergillosis in Post Tuberculosis Patients in Indonesia and the Role of LDBio Aspergillus ICT as Part of the Diagnosis Scheme | Journal of Fungi | Full text not available |
|  | Arage L.L, 2021 | Determinants of drug-induced hepatotoxicity among patients with human immunodeficiency virus taking a high dose of rifapentine plus isoniazid drugs at the all Africa leprosy tuberculosis rehabilitation and training center in Addis Ababa, Ethiopia | HIV/AIDS-Research and Palliative Care | Different populations  (HIV) |
|  | Baghaei P, 2011 | Adverse effects of multidrug-resistant tuberculosis treatment with a standardized regimen: a report from Iran | American journal of therapeutics | Report only descriptive findings |
|  | Balal M, 2005 | Loss of vision and renal function in a patient with miliary tuberculosis | Mount Sinai Journal of Medicine | Full text not available |
|  | Basu, S, 2014 | Degree, duration, and causes of visual impairment in eyes affected with ocular tuberculosis | Journal of Ophtalmic inflammation and infection | Report only descriptive findings |
|  | Batirel, A, 2015 | The course of spinal tuberculosis (Pott disease): results of the multinational, multicentre Backbone-2 study | Clinical Microbiology and Infection | Report treatment outcomes |
|  | Baig I.M 2010 | Post-tuberculous chronic obstructive pulmonary disease | Journal of the College of Physicians and Surgeons Pakistan | Report only descriptive findings |
|  | Bansal A, 2023 | Surviving Pulmonary Tuberculosis: Navigating the Long-Term Respiratory Effects | Cure us | Review |
|  | Bardien S. 2009 1996 | Aminoglycoside-induced hearing loss: South Africans at risk | South African Medical Journal | Editorial |
|  | Begum E, 2023 | Risk factors for hepatotoxicity in patients hospitalized for tuberculosis | European Journal of General Medicine | The outcome is not reported |
|  | Ben Fredj N. 2016 | Risk factors of isoniazid-induced hepatotoxicity in Tunisian tuberculosis | The Pharmacogenetics journal | Full text not available |
|  | Ben S. 2022 | Particularities of post-tuberculosis bronchiectasis | European Respiratory journal | Full text not available |
|  | Bhurayanontachai R 2016 | Factors influencing development and mortality of acute respiratory failure in hospitalized patient with active pulmonary tuberculosis: A 10-year retrospective review | Journal of Thoracic Disease | Study short-term outcomes and mortality |
|  | Binegdie, A. B. 2019 | Prevalence of Chronic Obstructive Pulmonary Disease (COPD) among patients successfully treated for pulmonary tuberculosis in Ethiopia | American Journal of Respiratory and Critical Care Medicine | Report only descriptive findings |
|  | Boloursaz MR 2023 | Radiologic Manifestation of Pulmonary Tuberculosis in Children Admitted in Pediatric Ward-Massih Daneshvari Hospital: A 5-Year Retrospective Study | Acta Medica Iranica | Full text not available |
|  | Bongomin F, 2023 | Post-tuberculosis chronic pulmonary aspergillosis: An emerging public health concern | PLoS Pathogens | Literature review |
|  | Bozzola E, 2020 | Predicting parameters for audiological complications in pediatric patients affected by meningitis | Journal of Pediatric Infectious Disease | Report only descriptive finding |
|  | Bright-Thomas RJ | Drug-related hepatitis in patients treated with standard antituberculosis chemotherapy over 30 years | International Journal of Tuberculosis and Lung Disease | Full text not available |
|  | Brits J 2023 | Hearing profile of gold miners with and without tuberculosis | Occupational and Environmental Medicine | Report only descriptive findings |
|  | Buziashvili, M 2019 | Rates and risk factors for nephrotoxicity and ototoxicity among tuberculosis patients in Tbilisi, Georgia | The International Journal of Tuberculosis and Lung Disease | Full text not available |
|  | Byrne A, 2017 | Chronic airflow obstruction after successful treatment of multidrug-resistant tuberculosis. | ERJ Open Research | Investigate treatment outcome |
|  | Chavan R.P, 2023 | Hearing assessment and treatment outcome in ENT tuberculosis at a tertiary hospital in India | The Egyptian Journal of Otolaryngology | Report only descriptive |
|  | Chee Y.C. 1981 | Ocular toxicity from ethambutol | Singapore Medical Journal | Review |
|  | Chen, H.L., 2015 | Structural deficits and cognitive impairment in tuberculous meningitis | BMC Infectious Disease | Report only descriptive findings |
|  | Chin, A. T 2019 | Chronic lung disease in adult recurrent tuberculosis survivors in Zimbabwe: A cohort study | International Journal of Tuberculosis and Lung Disease | Full text not available |
|  | Choi H, 2021 | Clinical characteristics of patients with post-tuberculosis bronchiectasis: Findings from the KMBARC registry | Journal of Clinical Medicine | Report descriptive findings only |
|  | Chong, GY 2019 | Prevalence of post-tuberculosis-chronic obstructive pulmonary disease at a tertiary referral center in Kuala Lumpur | Respirology | Full text not available |
|  | Chushkin M, 2012 | The factors influencing impairment of respiratory function in patients treated for pulmonary tuberculosis | Chest | Conference abstract |
|  | Chuenkongkaew W, 2003 | Ethambutol and optic neuropathy | Journal of the Medical Association of Thailand | Full text not available |
|  | Chushkin, M 2012 | The factors associated with pulmonary impairment in patients with treated tuberculosis | European Respiratory Society | Annual congress |
|  | Chushkin, M 2011 | Obstructive lung disease in patients with treated pulmonary tuberculosis | European Respiratory Society | Full text not available |
|  | Chushkin, M 2011 | The impairment of respiratory function in patients with treated pulmonary tuberculosis | Chest | Conference presentations |
|  | Coca N.S.M, 2010 | Antituberculosis drug-induced hepatotoxicity: a comparison between patients with and without human immunodeficiency virus seropositivity | Revista de Sociedade Brasileria de Medicina Tropical | Report only descriptive findings |
|  | De Jager 2002 | Hearing loss and nephrotoxicity in long-term aminoglycoside treatment in patients with tuberculosis | International Journal of Infectious Diseases | Full text not available |
|  | Demper J 2007 | Tuberculosis and phrenic nerve destruction | South Africa Medical Journal | Case report and series |
|  | Devoto, F.M. 1997 | Risk factors for hepatotoxicity induced by antituberculosis drugs | Acta Physiologica Pharmacologica et Therapeutica Latinoamericana | Full text not reported |
|  | Dhar R 2018 | Tuberculosis associated obstructive lung disease- A pilot study | European Respiratory Journal | Full text is not available |
|  | Dhawan S.R, 2016 | Predictors of neurological outcome of tuberculosis meningitis in childhood: A prospective cohort study from a developing country | Journal of Child Neurology | Studied treatment outcome |
|  | Doh H.K, 2008 | Treatment outcomes and long-term survival in patients with extensively drug-resistant tuberculosis | American Journal of Respiratory and Critical Care Medicine | Outcome not reported |
|  | Donald P, 1991 | Hearing loss in the child following streptomycin administration during pregnancy | Central Africa Journal of Medicine | Report only descriptive findings |
|  | Drosos A.A, 1985 | Tuberculous spondylitis: a cause for paraplegia in lupus | Rheumatology International | Case report and series |
|  | Dunn F.J, 2011 | Spinal tuberculosis: Magnetic resonance imaging and neurological impairment | Spine | Report only descriptive findings |
|  | Durand F, 1996 | Hepatotoxicity of antitubercular treatments. The rationale for monitoring liver status | Drug safety | Guideline |
|  | Egbagbe E 2008 | Ocular disorders in adult patients with tuberculosis in a tertiary care hospital in Nigeria | Middle East African Journal of Ophthalmology | Report only descriptive findings |
|  | Ergan B, 2017 | Risk factors for hepatotoxicity in patients hospitalized for tuberculosis | European Journal of General Medicine | Not enough information on comparison groups |
|  | Fernez-Villar, A 2004 | The influence of risk factors on the severity of anti-tuberculosis drug-induced hepatotoxicity | International Journal of Tuberculosis and Lung Disease | Full text not available |
|  | Fiogbe, A.A, 2019 | Prevalence of lung function impairment in cured pulmonary tuberculosis patients in Cotonou, Benin | International Journal of Tuberculosis and Lung Disease | Full text not available |
|  | Freimane L, 2023 | Assessment of Amikacin- and Capreomycin-related adverse drug reactions in patients with multidrug-resistant tuberculosis and exploring the role of genetic factors | Journal of Personalized Medicine | Studies short-term outcomes (adverse effects) |
|  | Gai X, 2003 | Post-tuberculosis lung disease and chronic obstructive pulmonary disease | Chinese Medical Journal | Review |
|  | Gandhi, K, 2016 | Risk factors associated with the development of pulmonary impairment after tuberculosis | Indian Journal of Tuberculosis | Full text not available |
|  | Garg P, 2015 | A prospective study of ocular toxicity in patients receiving ethambutol as a part of directly observed treatment strategy therapy | Lung India | Report only descriptive findings |
|  | Garg R.K, 2010 | Neurological complications of miliary tuberculosis | Clinical Neurology and Neurosurgery | Report only descriptive findings |
|  | Gehlot P, 2012 | Pott's spine: Retrospective analysis of MRI scans of 70 cases | Journal of Clinical and Diagnostic Research | No comparison group |
|  | Ghafari, N, 2015 | The occurrence of auditory dysfunction in children with TB receiving ototoxic medication at a TB hospital in South Africa | International Journal of Pediatric Otorhinolaryngology | Report only descriptive findings |
|  | Ghimire H 2011 | Impact of pulmonary tuberculosis infection on chronic obstructive pulmonary disease | European Respiratory Journal | Outcome not reported |
|  | Godoy, M DP, 2012 | The functional assessment of patients with pulmonary multidrug-resistant tuberculosis | Respiratory care | Report only descriptive findings |
|  | Golemba A. S, 2015 | Drug-induced hepatotoxicity and tuberculosis in a hospital from the Argentinian northeast: cross-sectional study | Medwave | Non-English |
|  | Gulliford M, 1986 | Cholestatic Jaundice Caused by Ethambutol | British Medical Journal (Clinical research ed.) | Case report and series |
|  | Gupta A 2017 | Pulmonary Tuberculosis: A Neglected Risk Factor for Deep Venous Thrombosis | International Journal Mycobacteriologyof Mycobacteriology | Outcome not reported |
|  | Gyawali S, 2023 | Previous tuberculosis infection associated with increased frequency of asthma and respiratory symptoms in a Nordic-Baltic multicentre population study | ERJ Open Research | Outcome not presented and not specific to TB |
|  | Ha KY, 2016 | Late onset of progressive neurological deficits in severe angular kyphosis related to tuberculosis | European Spine Journal | Reports only descriptive findings |
|  | Hegai L.N 2013 2015 | Chronic heart failure in patients with recurrent pulmonary tuberculosis | International journal of cardiology | Outcome not reported |
|  | Heysell SK, 2017 | Hearing loss with kanamycin treatment for multidrug-resistant tuberculosis in Bangladesh | The European Respiratory journal | Letter to the editor |
|  | Hiroi K, 1978 | Studies on pulmonary function in patients with pulmonary tuberculosis: —With Special Reference to Flow-Volume Curve and Closing Volume and Their Relation with Various Factors— | Kekkaku(Tuberculosis) | Non-English language |
|  | Hnizdo E, 2000 | Chronic pulmonary function impairment caused by initial and recurrent pulmonary tuberculosis following treatment | Thorax | Not specific to TB (Miners including TB patients) |
|  | Hong H 2018 | Increased risk of aminoglycoside-induced hearing loss in MDRTB patients with HIV coinfection | International Journal of Tuberculosis and Lung Disease | Full text not available |
|  | Hong H 2020 | Prevalence of Pre-Existing Hearing Loss Among Patients With Drug-Resistant Tuberculosis in South Africa | American Journal of Audiology | Full text not available |
|  | Hong H 2020 | Aminoglycoside induced hearing loss among patients being treated for drug-resistant tuberculosis in South Africa: A prediction model | Clinical Infectious Disease | The paper repeated by changing the statistical model |
|  | Honnorat E, 2013 | Encephalitis due to Mycobacterium tuberculosis in France | Medecine et Maladies Infectieuses | Report treatment outcome |
|  | Hotchandani H 2013 | Anti-Tuberculosis Therapy Induced Hepatotoxicity In Children | Pakistan Paediatric Journal | Full text not available |
|  | Hsia, N.Y, 2015 | Risk of cataract for people with tuberculosis: results from a population-based cohort study | International Journal of Tuberculosis and Lung Disease | Full text not available |
|  | Hussain Z 2003 | Antituberculosis drug-induced hepatitis: risk factors, prevention and management | Indian Journal of Experimental Biology | Review |
|  | Hwang Y, 2014 | The association between airflow obstruction and radiological change by tuberculosis | Journal of Thorax Disease | Reports only descriptive findings |
|  | Ina Jeong, 2015 | Drug-induced hepatotoxicity of anti-tuberculosis drugs and their serum levels | Journal of Korean Medical Science | Reports only descriptive findings |
|  | Jagmohan HV, 2022 | "Prevalence of chronic obstructive pulmonary disease in tuberculosis and vice-versa in a tertiary care hospital" | Journal of Cardiovascular Disease Research | Full text not available |
|  | Jimenz C, 2012 | Association of Diabetes and tuberculosis: impact on treatment and post-treatment outcomes | Thorax | Outcome not reported |
|  | Jimenz L, 1987 | Isoniazid and ethambutol as a cause of optic neuropathy | Europe PMC | Full text not available |
|  | Jing C, 2023 | Disease burden of tuberculosis and post-tuberculosis in Inner Mongolia, China, 2016-2018 - based on the disease burden of post-TB caused by COPD | BMC Infectious Disease | Report only descriptive findings |
|  | Kabir SK, 2023 | Assessment of the hepatotoxicity induced by anti-tuberculosis drugs in tuberculosis subjects undergoing treatment | Journal of Cardiovascular Disease Research | Reports only descriptive findings |
|  | Kamenar K, 2020 | Previous tuberculosis disease as a risk factor for chronic obstructive pulmonary disease: a cross-sectional analysis of multicountry, population-based studies | Thorax | Not specific to TB |
|  | Kanesen D, 2021 | Clinical outcome of tuberculous meningitis with hydrocephalus — a retrospective study | Malaysian Journal of Medical Sciences | Outcome not reported |
|  | Karande, S. 2005 | Prognostic clinical variables in childhood tuberculous meningitis: an experience from Mumbai, India | Neurology Society of India | Full text not available |
|  | Karuppannasamy D, 2014 | Linezolid-induced optic neuropathy | Indian Journal of Ophthalmology | Case report and series |
|  | Keshavjee, S. 2012 | Hepatotoxicity during treatment for multidrug-resistant tuberculosis: occurrence, management and outcome | International Journal of Tuberculosis and Lung Disease | Full text not available |
|  | Kewalramani M.S, 2020 | Evaluation of hepatotoxicity of anti-tuberculosis regimens: A prospective study in Tribal population of Central India | Journal of Young Pharmacists | Report only descriptive findings |
|  | Khan R, 2023 | Imaging of Pulmonary Post-Tuberculosis Sequelae | Pakistan Journal of Medical Sciences Quarterly | Review |
|  | Khoza SK, 2023 | Audiological testing for ototoxicity monitoring in adults with tuberculosis in state hospitals in Gauteng, South Africa | Southern African Journal of Infectious Diseases | Report only descriptive findings |
|  | Kim T, 2021 | Respiratory symptoms and health-related quality of life in post-tuberculosis subjects with physician-diagnosed bronchiectasis: A cross-sectional study | Journal of Thoracic Disease | Report only descriptive findings |
|  | Kolur S.S, 2023 | Impairments in pulmonary functions in pediatric spinal tuberculosis: a cross-sectional study | Spine Deformity | Report only descriptive findings |
|  | Kumar R, 2020 | Identification of risk factors for radiological sequelae in patients treated for pulmonary tuberculosis: Prospective observational cohort study | Indian Journal of Tuberculosis | Non-English language and no full text not available |
|  | Kumar S, 2016 | Prevalence and outcome of headache in tuberculous meningitis | Neurosciences | Report treatment outcome |
|  | Kwon, Y.S. 2023 | Risk factors for peripheral neuropathy in patients on linezolid-containing regimens for drug-resistant TB | International Journal of Tuberculosis and Infectious Disease | Full text not available |
|  | Lam H, 2010 | Prior TB, smoking, and airflow obstruction: a cross-sectional analysis of the Guangzhou Biobank Cohort Study | Chest | Not specific to TB (includes general population |
|  | Lee, A.M, 2002 | Risk factors for hepatotoxicity associated with rifampin and pyrazinamide for the treatment of latent tuberculosis infection: Experience from three public health tuberculosis clinics | International Journal of Tuberculosis and Lung Disease | Studied latent TB cases |
|  | Lee L.N, 2014 | PXR modulates hepatotoxicity during anti-tuberculosis treatment: Sex matters | American Journal of Respiratory and Critical Care Medicine | Full text not available |
|  | Lees A.W, 1971 | Toxicity from rifampicin plus isoniazid and rifampicin plus ethambutol therapy | Tubercle | Case report and series |
|  | Lee, J.H. 2003 | Lung function in patients with chronic airflow obstruction due to tuberculous destroyed lung | Respiratory Medicine | Report only descriptive findings |
|  | Lima M | Hearing impairment in patients with tuberculosis from Northeast Brazil | Rev.Inst.Med.Trop.S.Paulo | Case series |
|  | Maguire G.P, 2009 | Pulmonary tuberculosis, impaired lung function, disability, and quality of life in a high-burden setting | International Journal of Tuberculosis and Infectious Disease | Report only descriptive findings |
|  | Mancuzo E.V, 2019 | Spirometry results after treatment for pulmonary tuberculosis: comparison between patients with and without previous lung disease: a multicenter study | Jornal brasileiro de pneumologia : publicacao oficial da Sociedade Brasileira de Pneumologia e Tisilogia | Report only descriptive findings |
|  | Mankhatitham W, 2010 | Hepatotoxicity in patients co-infected with HIV and tuberculosis while receiving NNRTI-based antiretroviral regimen and rifampicin | Journal of the International AIDS Society | Conference abstract |
|  | Marcos P 2011 | Long-term follow-up in patients diagnosed and treated for pulmonary tuberculosis: What happened with the lung function? | Chest | Conference abstract |
|  | Maria N, 2017 | The Impact of antituberculosis drug-induced hepatotoxicity to successful tuberculosis treatment in Indonesia | Asian Journal of Pharmaceutical and Clinical Research | Outcome not presented |
|  | Matsson K 1974 | Acute renal failure following rifampicin administration | Scandinavian Journal of Respiratory Diseases | Case report and series |
|  | Meghji. J 2019 | Patient outcomes associated with post-tuberculosis lung damage in Malawi: A prospective cohort study | Thorax | Outcome not reported |
|  | Menon B, 2015 | Evaluation of the radiological sequelae after treatment completion in new cases of pulmonary, pleural, and mediastinal tuberculosis | Lung India | Report descriptive findings only |
|  | Miftode E.G, 2015 | Tuberculous Meningitis in Children and Adults: A 10-Year Retrospective Comparative Analysis | PLOS ONE | Outcome not reported |
|  | Mkoko P, 2019 | Chronic lung disease and a history of tuberculosis (Post-tuberculosis lung disease): Clinical features and in-hospital outcomes in a resource-limited setting with a high HIV burden | South African Medical Journal | Outcome not reported |
|  | Modongo C, 2015 | Amikacin concentrations predictive of ototoxicity in multidrug-resistant tuberculosis patients | Antimicrobial Agents and Chemotherapy | Review |
|  | Mooney, A. J., 1956 | Some ocular sequelae of tuberculous meningitis: A preliminary survey, 1953–1954 | American Journal of Ophthalmology | Editorial |
|  | Nabukeera-Barungi, N, 2014 | Presentation and outcome of tuberculous meningitis among children: experiences from a tertiary children's hospital | African Health Sciences | Report only descriptive findings |
|  | Nadr L.A, 2010 | Hepatotoxicity due to rifampicin, isoniazid, and pyrazinamide in patients with tuberculosis: is anti-HCV a risk factor? | Annals of hepatology | Report only descriptive findings |
|  | Namusobya M, 2022 | Chronic pulmonary aspergillosis in patients with active pulmonary tuberculosis with persisting symptoms in Uganda | Mycoses | Outcome not reported |
|  | Nataprawira, H.M. 2016 | Outcome of tuberculous meningitis in children: the first comprehensive retrospective cohort study in Indonesia | International Journal of Tuberculosis and Lung Disease | Full text not available |
|  | Need a.g, 1980 | Rifampicin-associated renal dysfunction during antituberculous therapy | Australian and New Zealand journal of medicine | Case report and series |
|  | Ngoc T.B, 2023 | Chronic Pulmonary Aspergillosis Situation among Post Tuberculosis Patients in Vietnam: An Observational Study | Journal of Fungi | Report only descriptive findings |
|  | Nightingale R, 2021 | Respiratory symptoms and lung function in patients treated for pulmonary tuberculosis in Malawi: a prospective cohort study | Thorax | Outcome not presented |
|  | Nijoku, C.H. 2007 | Experiences in management of Pott's paraplegia and paraparesis in medical wards of Usmanu Danfodiyo University Teaching Hospital, Sokoto, Nigeria | Annals of African Medicine | Report only descriptive findings |
|  | Nishi M P, 2021 | Pulmonary functional assessment: longitudinal study after treatment of pulmonary tuberculosis | Revista do Instituto de Medicina Tropical de São Paulo | Report only descriptive findings |
|  | O’Brien, R.J. 1983 | Hepatotoxicity from isoniazid and rifampin among children treated for tuberculosis | Pediatrics | Report only descriptive findings |
|  | O’Connor, B. 2014 | The prevalence of chronic obstructive pulmonary disease in patients with culture-confirmed pulmonary tuberculosis | Irish Journal of Medicinal Science | Annual Scientific meetings |
|  | Ohkawa K, 2002 | Risk factors for anti-tuberculosis chemotherapy-induced hepatotoxicity in Japanese pediatric patients | Clinical pharmacology and therapeutics | Methodological not appropriate |
|  | Osman, R.K. 2016 | Chronic respiratory disease in adults treated for tuberculosis in Khartoum, Sudan | Public Health Action | Outcome not reported |
|  | Panda, A, 2016 | Correlation of chest computed tomography findings with dyspnea and lung functions in post-tubercular sequelae | Lung India | Report only descriptive findings |
|  | Park D.H. 2015 | Linezolid induced retinopathy | Documenta Ophthalmologica | Case report and series |
|  | Park, H.J. 2018 | History of pulmonary tuberculosis affects the severity and clinical outcomes of COPD | Respirology | Report another outcome (Effect of TB on COPD patients) |
|  | Pasipanodya J. G, 2023 | Pulmonary impairment after tuberculosis and its contribution to TB burden | BMC Public Health | Report treatment outcome |
|  | Patil, S.V, 2007 | Cardiac dysfunction in active pulmonary tuberculosis: Mysterious facts of TB’s pandora | Electronic Journal of General Medicine | Reports only descriptive findings |
|  | Pietersen E, 2023 | High Frequency of Resistance, Lack of Clinical Benefit, and Poor Outcomes in Capreomycin Treated South African Patients with Extensively Drug-Resistant Tuberculosis | PLOS ONE | Report treatment outcome |
|  | Piparva, K, G, 2018 | Evaluation of treatment outcome and adverse drug reaction of directly observed treatment (DOT) plus regimen in multidrug-resistant tuberculosis (MDR-TB) patients at district tuberculosis center Rajkot | Perspectives in Clinical Research | Report only descriptive findings |
|  | Pore S, 2023 | Risk factors for drug-induced hepatitis with first line antituberculosis drugs in hospitalized patients of pulmonary tuberculosis | Journal of Postgraduate Medicine | Editorial |
|  | Prasad, R. 2006 | Predisposing factors in hepatitis induced by anti-tuberculosis regimens containing isoniazid, rifampicin, and pyrazinamide: A case-control study | Journal of Internal Medicine of India | Report only descriptive findings |
|  | Qureshi, U.A, 2020 | Determine the hepatotoxicity with anti-tuberculosis drugs and its severity and frequency | Pakistan Journal of Medical and Health Sciences | Reports only descriptive findings |
|  | Radovic M, 2011 | Chronic airflow obstruction syndrome due to pulmonary tuberculosis treated with directly observed therapy--a serious change in lung function | Medicinski arhiv | Conferences abstract |
|  | Shakya R, 2004 | Incidence of hepatotoxicity due to antitubercular medicines and assessment of risk factors | The Annals of Pharmacotherapy | Only 13 samples (4 cases) |
|  | Ramma, Ld Fau, | Efficacy of utilizing patient self-report of auditory complaints to monitor aminoglycoside ototoxicity | International Journal of Tuberculosis and Lung Disease | Full text not available |
|  | Ramos, L.M. 2005 | Functional profile of patients with tuberculosis sequelae in a university hospital | Journal of Brasileiro de Pneumologia | Report only descriptive findings |
|  | Rehman N, 2019 | Prevalence of anti-tuberculosis drug-induced hepatitis in patients on anti-tuberculous drug in Mardan medical complex | Medical Journal Monthly | Conference abstract |
|  | Revendran, J 2018 | Long-term sequelae of pulmonary tuberculosis and the factors predicting its development: A clinicopathological study | European Respiratory Journal | Full text not available |
|  | Ruan H, 2022 | Risk factors for respiratory failure after tuberculosis-destroyed lung surgery and increased dyspnea score at 1-year follow-up | Journal of Thoracic Disease | Different populations (evaluate effect after surgery) |
|  | Ruiz ML, 2016 | Tuberculous spondylitis (pott's disease) a common cause of paraparesis in young Mexicans, its association with other forms of tuberculosis, and the use of MRI as a diagnostic tool | Neurology | Letter to the editor |
|  | Russo P, 1994 | Toxic optic neuropathy associated with ethambutol: implications for current therapy | Europe PMC | Full text not available |
|  | Ryoo J, 2023 | Prognostic factor of respiratory failure in tuberculosis | Intensive Care Medicine Experiment | Meeting abstracts |
|  | Ryoo, J, 2023 | Isoniazid-resistant tuberculosis is associated with hepatotoxicity: A prospective multicenter cohort study of pulmonary tuberculosis | Respirology | Full text not available |
|  | Sabur N.F, 2023 | Low-dose amikacin in the treatment of Multidrug-resistant Tuberculosis (MDR-TB) | BMC Infectious Disease | Assess treatment outcome |
|  | Sagwa, E.L. 2017 | Renal function of MDR-TB patients treated with kanamycin regimens or concomitantly with antiretroviral agents | International Journal of Tuberculosis and Lung Disease | Full text not available |
|  | Santra, A. 2017 | Clinico-Radiologic and Spirometric Profile of an Indian Population with Post-Tuberculous Obstructive Airway Disease | Journal of Clinical and Diagnostic Research | Report only descriptive findings |
|  | Saroha D 2020 | Irreversible neuropathy in extremely-drug resistant tuberculosis: An unfortunate clinical conundrum | The Lancet | Case report |
|  | Seufert C.D. 1973 | Acute renal failure after rifampicin therapy | Scandinavian journal of respiratory diseases | Full text not available |
|  | Saukkonen J.J,2006 | An official ATS statement: hepatotoxicity of antituberculosis therapy | American journal of respiratory and critical care medicine | Literature review |
|  | Saylor D, 2021 | Neurologic Complications of Tuberculosis | CONTINUUM Lifelong Learning in Neurology | Review |
|  | Seddon, J.A. 2012 | Hearing loss in patients on treatment for drug-resistant tuberculosis | European Respiratory Journal | Review |
|  | Sehgal I, S, 2023 | Burden, clinical features, and outcomes of post-tuberculosis chronic obstructive lung diseases | Current opinion in pulmonary medicine | Report only descriptive findings |
|  | Shaarawy, H, 2018 | Assessment of the prevalence of pulmonary embolism in patients with severe pulmonary tuberculosis | Egyptian Journal of Chest Diseases and Tuberculosis | Report only descriptive findings |
|  | Shaikh MA 2022 | Frequency of hepatotoxicity during anti-tuberculous treatment at a medical unit of LUMHS Sindh | Medical Channel | Full text not available |
|  | Shang P, 2011 | Incidence, Clinical Features and impact on Anti-tuberculosis Treatment of anti-tuberculosis drug-induced Liver Injury (ATLI) in China | PLOS ONE | Report only descriptive findings |
|  | Shanmugam MK 2022 | Ethambutol induced toxic optic neuropathy - A retrospective study in a tertiary eye care center in Southern India | Nepalese journal of ophthalmology | Full text not available |
|  | Shean, K. 2013 | Drug-associated adverse events and their relationship with outcomes in patients receiving treatment for extensively drug-resistant tuberculosis in South Africa | PLOS ONE | Report mortality and adverse events |
|  | Shen, X. 2013 | Anti-tuberculosis drug-induced liver injury in Shanghai: validation of Hy's Law | Drug safety | Full text not available |
|  | Shibeshi, W, 2019 | Nephrotoxicity and ototoxic symptoms of injectable second-line anti-tubercular drugs among patients treated for MDR-TB in Ethiopia: a retrospective cohort study | BMC Pharmacology and Toxicology | Report only descriptive findings |
|  | Shin HJ, 2003 | Peripheral neuropathy associated with treatment for multidrug-resistant tuberculosis | International Journal of Tuberculosis and Lung Disease | Full text not available |
|  | Shin HJ 2023 | Hepatotoxicity of anti-tuberculosis chemotherapy in patients with liver cirrhosis | International Journal of Tuberculosis and Lung Disease | Full text is not available |
|  | Singla N, 2009 | Post-treatment sequelae of multi-drug resistant tuberculosis patients | Indian Journal of Tuberculosis | Editorial |
|  | Singla, R. 2017 | Sequelae of pulmonary multidrug-resistant tuberculosis after treatment | Indian Chest Society | Report only descriptive findings |
|  | Smith J, 1972 | Hepatotoxicity in rifampin-isoniazid-treated patients related to their rate of isoniazid inactivation | Chest | Full text not available |
|  | Song JH, 2023 | The clinical impact of drug-induced hepatotoxicity on anti-tuberculosis therapy: a case-control study | Respiratory Research | Studies different outcome |
|  | Subbalaxim 2020 | Evaluation of Risk Factors for Development of Anti-Tubercular Therapy Induced Hepatotoxicity: A Prospective Study | Current drug safety | Full text is not available |
|  | Subhadrabandhu T 1992 | Risk factors for neural deficit in spinal tuberculosis | Journal of the Medical Association of Thailand | Full text is not available |
|  | Sugita H, 1983 | Clinical Study on Respiratory Failure Due to Tuberculosis | Kekkaku(Tuberculosis) | Non-English language |
|  | Sun-Hyung K, 2023 | Clinical Factors Associated with Cavitary Tuberculosis and Its Treatment Outcomes | Journal of Personalized Medicine | Outcome not reported |
|  | Talbert Estlin K.A, 2010 | Risk factors for ethambutol optic toxicity | International Ophthalmology | Systematic review |
|  | Tajender V, 2006 | INH-induced status epilepticus: response to pyridoxine | The Indian journal of chest diseases & allied sciences | Full text is not available |
|  | Teleman MD, 2002 | Hepatotoxicity of tuberculosis chemotherapy under general program conditions in Singapore | International Journal of Tuberculosis and Lung Disease | Full text is not available |
|  | Thorson A, 2007 | Chest X-ray findings about gender and symptoms: A study of patients with smear-positive tuberculosis in Vietnam | Scandinavian Journal of Infectious Diseases | Reports only descriptive findings |
|  | Tost J.R, 2005 | Severe hepatotoxicity due to anti-tuberculosis drugs in Spain | International Journal of Tuberculosis and Lung Disease | Full text not available and outcome not reported |
|  | Tuli, S.M, 1995 | Severe kyphotic deformity in tuberculosis of the spine | International Orthopaedics | Reported only descriptive findings |
|  | Tweed, C.D, 2016 | Liver function tests during tuberculosis treatment and the implications on monitoring for hepatotoxicity | Thorax | Conference presentations |
|  | Tweed C.D, 2018 | Toxicity associated with tuberculosis chemotherapy in the REMoxTB study | BMC Infectious disease | Report adverse outcomes |
|  | Umair-UL, 2019 | Determine the hepatotoxicity with anti-tuberculosis drugs and its severity and frequency | Pakistan Journal of Medical and Health Sciences | Report only descriptive findings |
|  | Urzra C.A, 2016 | Clinical Features and Prognostic Factors in Presumed Ocular Tuberculosis | Current eye research | Outcome not related/no multivariable analysis |
|  | Van Altena. R | Reduced chance of hearing loss associated with therapeutic drug monitoring of aminoglycosides in the treatment of multidrug-resistant tuberculosis | Antimicrobial Agents and Chemotherapy | Report treatment adverse outcomes |
|  | Van Hest, 2004 | Hepatotoxicity of rifampin-pyrazinamide and isoniazid preventive therapy and tuberculosis treatment | Clinical Infectious Diseases | Studies LTBI not active TB |
|  | Van Kampen, S.C, 2019 | Chronic Respiratory Symptoms and Lung Abnormalities among People with a History of Tuberculosis in Uganda: A National Survey | Clinical Infectious Diseases | Not specific to TB (Including the general population) |
|  | Vashakidze S, 2018 | Pulmonary function and respiratory health after successful treatment of drug-resistant tuberculosis | International Journal of Infectious Disease | Reports only descriptive findings |
|  | Vasudeva, R. 1997 | Isoniazid-related hepatitis | Digestive diseases (Basel, Switzerland) | Report only descriptive findings |
|  | Vasudevan D, 2021 | Assessment of Pulmonary Function Test in Patients Having Tuberculosis, Asthma and COPD | European Journal of Molecular and Clinical Medicine | Full text is not available |
|  | Vecino M, 2011 | Evidence for chronic lung impairment in patients treated for pulmonary tuberculosis | Journal of infection and public health | Reports only descriptive findings |
|  | Vijayalakshmi, A 2016 | A prospective study on abnormal liver function test patterns in patients receiving anti-tuberculosis therapy. | Asian Journal of Pharmaceutical and Clinical Research | Report only descriptive findings |
|  | Wang, Y.2022 | Impact of previous pulmonary tuberculosis on chronic obstructive pulmonary disease: Baseline Results from a Prospective Cohort Study | Combinatorial chemistry and high throughput screening | Outcome not reported |
|  | Wangchuk P, 2021 | Audiological monitoring of patients undergoing multidrug-resistant tuberculosis treatment at Jigme Dorji Wangchuk National Referral Hospital and Gidakom Hospital, Bhutan | journal of Clinical Tuberculosis and Other Mycobacterial Diseases | Reports only descriptive findings |
|  | Warmelink I, 2011 | Weight loss during tuberculosis treatment is an important risk factor for drug-induced hepatotoxicity | British Journal of Nutrition | Outcome not reported (Studied reasons for interruption) |
|  | Wasserman S, 2022 | Linezolid toxicity in patients with drug-resistant tuberculosis: A prospective cohort study | Journal of Antimicrobial Chemotherapy | No report on OR, RR, HR, or two-by-two table |
|  | Willcox, P.A, 1989 | Chronic obstructive airways disease following treated pulmonary tuberculosis | Respiratory Medicine | Reports only descriptive findings |
|  | Wen P, 2019 | Risk factors for tuberculous empyema in pleural tuberculosis patients | Scientific Reports | Report acute complications (Empeyma) |
|  | Wiman L. 1974 | Amyloidosis and lung diseases | Scandinavian journal of respiratory diseases | Full text is not available |
|  | Xing Z, 2022 | Airflow obstruction and small airway dysfunction following pulmonary tuberculosis: a cross-sectional survey | Thorax | Not specific to TB (Including the general population) |
|  | Yaker H. I, 2017 | The role of tuberculosis in COPD | International Journal of COPD | Report mortality |
|  | Yang H.K. 2016 | Incidence of toxic optic neuropathy with low-dose ethambutol | International Journal of Tuberculosis and Lung Disease | Full text is not available |
|  | Yeyung L. 2022 | High prevalence of COPD despite successful tuberculosis treatment | European respiratory journal | Full text is not available |
|  | Yew W.W, 2001 | Risk factors for hepatotoxicity during anti-tuberculosis chemotherapy in Asian populations | International Journal of Tuberculosis and Lung Disease | Full text is not available |
|  | Yimer G, 2023 | Evaluation of Patterns of Liver Toxicity in Patients on Antiretroviral and Anti-Tuberculosis Drugs: A Prospective Four-Arm Observational Study in Ethiopian Patients | PLOS ONE | The comparison group is not clearly shown in the multivariable analysis |
|  | Zhang Z, 2023 | The outcomes of chemotherapy-only treatment on mild spinal tuberculosis | Journal of Orthopaedic Surgery and Research | Reports only descriptive findings |
|  | Zhao H, 2020 | Drug-induced liver injury from anti-tuberculosis treatment: A retrospective cohort study | Medical Science Monitor | Reports only descriptive findings |
|  | Zifodya, J.S. 2023 | Post tuberculosis lung disease among Kenyan adults: an interim analysis | American Journal of the Medical Sciences | Full text not available |

**Supplementary file: Table 3**: Narrative synthesis for the risk factors of lung sequelae.

| **Risk factors** | **Number of studies** | **Direction of association (study reference)** |
| --- | --- | --- |
| **Socio-demographic characteristics** | | |
| Older age | 9 | Risk (1-5) |
|  |  | Not significant(6-9) |
| Male gender | 16 | Risk (3, 7, 10-12) |
|  |  | Preventive (6) |
|  |  | Not significant (2, 5, 8, 9, 12-17) |
| BMI <18.5 | 6 | Risk (7, 18) |
|  |  | Not significant (5, 6, 9, 12) |
| Income | 1 | Risk (11) |
| Having a paid job | 1 | Not significant (4) |
| Low education level | 1 | Risk (2) |
| White skin color | 1 | Not significant (17) |
| Poverty | 1 | Not significant (16) |
| **Patient’s behavior** | | |
| Ever smoked | 15 | Risk (10, 11, 19) |
|  |  | Not significant (2, 4-6, 8, 9, 13, 14, 16-18, 20) |
| Current smoker | 3 | Preventive (18) |
|  |  | Not significant (5, 13) |
| Alcohol drinker | 3 | Risk (21) |
|  |  | Not significant (4, 9) |
| Number of packed smoked | 1 | Protective (9) |
| **TB history and treatment** | | |
| Previous TB history | 6 | Risk (1-4, 20, 21) |
| Longer duration of illness | 4 | Risk (22) |
|  |  | Preventive (14) |
|  |  | Not significant (6, 23) |
| Pseudomonas in culture | 1 | Not significant (14) |
| Smear positive | 2 | Risk (2, 24) |
| Sputum conversion rate | 1 | Risk (25) |
| Smear grading +1 | 1 | Not significant (6) |
| Smear grading +2 | 1 | Not significant (6) |
| **Comorbid conditions** | | |
| HIV positive | 5 | Preventive (3) |
|  |  | Not significant (4, 9, 12, 22) |
| CD4 Count<200 | 2 | Not significant (9, 13) |
| HBA1C | 1 | Not significant (13) |
| Diabetics mellitus | 3 | Risk (5, 6) |
|  |  | Not significant (24) |
| Presence of multiple diseases | 1 | Not significant (19) |
| Presence of cardiovascular disease | 1 | Risk (23) |
| Immune suppressive condition | 1 | Risk (26) |
| **Radiological features** | | |
| Pulmonary lesions on radiology | 6 | Risk (10, 15, 22, 23, 25) |
|  |  | Not significant (6) |
| Radiological score >8 | 1 | Not significant (22) |
| Overall affected lung/extent | 2 | Not significant (8, 9) |
| Radiological thickening | 1 | Not significant (27) |
| **Presence of clinical symptoms** | | |
| Duration of dyspnea retained | 1 | Risk (1) |
| Longer time has elapsed since the last TB | 1 | Preventive (1) |
| Number of exacerbations | 1 | Risk (1) |
| Recurrent cold cough | 1 | Preventive (14) |
| Current respiratory symptoms | 1 | Risk (20) |
| Chronic respiratory symptoms | 1 | Risk (20) |
| Longer duration of symptoms | 1 | Risk (22) |
| Phlegm | 1 | Not significant (22) |
| Night sweating | 1 | Risk (4) |
| **Environmental related conditions** | | |
| Separate kitchen from the main house | 1 | Not significant (26) |
| Outdoor Kitchen | 1 | Not significant (26) |
| A heating system in the house | 1 | Not significant (4) |
| Main cooker users | 1 | Not significant (4) |
| **Dietary habits** | | |
| Inadequate food | 1 | Not significant (4) |
| Eating > two meals per day | 1 | Not significant |
| Having cereals more than once a week | 1 | Risk (4) |
| Having fruits more than once a week | 1 | Preventive (4) |
| Having greasy food more than once a week | 1 | Risk (4) |
| Taking juice more than once a week | 1 | Not significant (4) |
| Having sweats more than once a week | 1 | Not significant (4) |

***BMI****: Body Mass Index,* ***CD4****: Cluster of Differentiation,* ***HBAIC****: Hemoglobin AIC,* ***HIV****: Human Immune Deficiency Virus, and* ***TB****: Tuberculosis*

**Supplementary file: Table 4**: Narrative synthesis for the risk factors of post-TB liver injury among patients treated for tuberculosis.

| **Risk factors** | **Number of studies** | **Direction of association** |
| --- | --- | --- |
| **Socio-demographic characteristics** | | |
| Male gender | 12 | Risk (28, 29) |
|  |  | Preventive (30) |
|  |  | Not significant(31-39) |
| Older age | 7 | Risk (36, 40) |
|  |  | Not significant(31, 32, 34, 37, 39) |
| BMI<18.5 | 5 | Risk (31, 41, 42) |
|  |  | Preventive (43) |
|  |  | Not significant(39) |
| **Patient behavior** | | |
| Chronic alcoholism | 6 | Not significant(28, 31, 34, 40, 44, 45) |
| Illicit ever drug user | 2 | Not significant(31, 34) |
| Polydrug intake | 1 | Risk (46) |
| Smoking | 2 | Not significant(33, 34) |
| **TB treatment and history** | | |
| Extrapulmonary TB compared to pulmonary | 3 | Risk (42, 47) |
|  |  | Not significant(41) |
| Disseminated TB compared to pulmonary-only TB | 3 | Not significant(39, 41, 42) |
| Pulmonary TB | 3 | Preventive (40) |
|  |  | Not significant(33, 35) |
| RIPE regimen | 1 | Not significant |
| Previous TB treatment | 2 | Risk (32) |
|  |  | Not significant(45) |
| Longer treatment duration (>=20 months) | 1 | Risk (32) |
| Mixed or daily dosing schedule | 1 | Not significant(44) |
| Rifampin daily dosage of>=600mg | 1 | Risk |
| Shorter TB treatment regimen | 1 | Not significant(34) |
| **Clinical conditions** | | |
| WHO stage 4 (HIV patients) | 1 | Not significant(41) |
| CD4 <200 | 3 | Risk (31) |
|  |  | Not significant(34, 41) |
| Chronic liver disease | 3 | Risk (39) |
|  |  | Preventive (43) |
|  |  | Not significant(28) |
| Hepatitis B co-infection | 5 | Risk (44, 48) |
|  |  | Not significant(28, 31, 46) |
| Hepatitis C | 4 | Risk(38) |
|  |  | Not significant (31, 46) |
|  |  | Risk(49) |
| Hepatitis B or C | 2 | No association(34, 49) |
| Initiated HAART before TB | 2 | Not significant(31, 34) |
| Initiated HAART during the 1st two months of treatment | 1 | Not significant(31) |
| 2NRTI + 1NNRTI | 1 | Not significant(31) |
| 2NRTI + 1PI/R | 1 | Not significant(31) |
| Viral load<100,000 copies/mm3 | 1 | Not significant(34) |
| Not on ART | 1 | Not significant(34) |
| Other opportunistic infections | 1 | No association (34) |
| No pre-existing hepatic disease | 1 | Preventive (34) |
| No Pyrazinamide for extensive disease | 1 | Risk(50) |
| Using Pyrazinamide for extensive disease | 1 | Not significant(50) |
| Malnutrition | 1 | Not significant(48) |
| HIV positive | 6 | Risk (30, 39, 46-48) |
|  |  | Not significant(45) |
| Presence of metabolic disorder | 1 | Risk (37) |
| Cotrimoxazole use | 1 | Not significant(38) |
| Presence of comorbidity | 1 | Risk (40) |
| Lung transplantation | 1 | Risk (51) |
| Kidney transplantation | 1 | Preventive (51) |
| Autoimmune disease | 1 | Risk (30) |
| Severe CKD without hemodialysis | 1 | Risk (52) |
| High initial hepatitis B viral load | 1 | Risk (52) |
| High initial Hepatitis viral load | 1 | Not significant(52) |
| Hypo-albuminemia | 4 | Risk (37, 39, 53) |
|  |  | Preventive (48) |
| 2FDC-HRZE half/4FDC-RH compared 2HRZE/4RH | 1 | Risk (29) |

***BMI****: Body Mass Index,* ***CD4****: Cluster of Differentiation, CKD: Chronic Kidney Disease, FDC: Fixed Dose Combination, HAART: Highly Active Antiretroviral Therapy,* ***HIV****: Human Immune Deficiency Virus, NRTI: Nucleoside Reverse Transcriptase Inhibitors, NNRTI: Non-Nucleoside Reverse Transcriptase Inhibitors RH: Rifampicin and Isoniazid, PI: Protease Inhibitors, RIPE: Rifampicin plus Isoniazid plus pyrazinamide plus ethambutol,* ***TB****: Tuberculosis, and WHO: World Health Organization*

**Supplementary file: Table 5:** Narrative synthesis for the risk factors of hearing sequelae among included studies

| **Factors** | **Number of studies** | **Direction of association** |
| --- | --- | --- |
| **Participants characteristics** | | |
| Male gender | 3 | Risk (32, 54) |
|  |  | Not significant(55) |
| BMI on admission | 1 | Preventive (56) |
| Older age | 2 | Not significant (32, 54) |
| **TB history and treatment** | | |
| Previous TB treatment | 1 | Risk (32) |
| Longer treatment duration (>=20 months) | 1 | Risk (32) |
| Pyrazinamide containing regimens | 1 | Risk (44) |
| Regimens other than Pyrazinamide, Rifmbicin, and Isonized | 1 | Risk (44) |
| Weekly aminoglycoside dose (>=75mg/kg/body compared to <75) | 1 | Risk (57) |
| Type of aminoglycoside Kanamycin compared with Amikacin | 1 | Not significant(57) |
| Amikacin-based regimen | 1 | Not significant(55) |
| Pre-XDR or XDR-TB | 1 | Not significant(55) |
| Extrapulmonary involvement | 1 | Not significant(55) |
| Positive culture results | 1 | Risk(55) |
| **Comorbidity conditions** | | |
| Type of audiometry (Kudowave) | 1 | Not significant(57) |
| HIV positive | 5 | Risk (54-56, 58, 59) |
| Presence of diabetes | 2 | Not significant (56, 60) |
| Hepatitis | 1 | Risk (44) |
| Baseline hearing problem | 2 | Risk (57) |
|  |  | No association (44) |

***BMI****: Body Mass Index,* ***TB****: Tuberculosis, and* ***XDR****: Extensively Drug-Resistant*

**Supplementary file: Table 6:** Narrative synthesis for the risk factors of neurological sequelae

| **Factors** | **Number of studies** | **Directions of association** |
| --- | --- | --- |
| **Participants characteristics** | | |
| BMI>25 | 1 | Risk (61) |
| Age >30 years | 1 | Risk (32) |
| **TB history and treatment** | | |
| Previous TB treatment | 1 | Not significant(32) |
| Longer treatment duration (>=20 months) | 1 | Risk (32) |
| **Spinal related characteristics** | | |
| Canal encroachment >50% | 1 | Risk (62) |
| Kyphosis>30 | 1 | Risk (62) |
| Middle thoracic | 1 | Not significant(61) |
| Distal thoracic | 1 | Not significant(61) |
| Pan vertebral involvement | 1 | Not significant(61) |
| Loss posterior CSF | 1 | Not significant(61) |
| Spinal cord cross-sectional area ratio > 45% | 1 | Not significant(63) |
| Cord signal change | 1 | Risk (61) |
| Spinal cord sagittal diameter ratio > 47% | 1 | Not significant (63) |
| Spinal cord angle < 95° | 1 | Not significant(63) |
| Preoperative sagittal Cobb angle > 104° | 1 | Not significant(63) |
| Segments involved > 5 | 1 | Not significant(63) |
| Time to spinal deformity per year | 1 | Not significant(63) |
| Spinal cord signal change | 1 | Not significant(63) |
| Kyphosis correction rate > 56% | 1 | Not significant(63) |
| Intraoperative neuromonitoring events ≥ 1 | 1 | Not significant(63) |
| Estimated blood loss > 1745 ml | 1 | Not significant(63) |
| Operation time > 325 min | 1 | Not significant(63) |
| level of consciousness (Good) | 1 | Preventive (64) |
| Focal motor deficit | 1 | Risk (64) |
| **Comorbidity condition** | | |
| HIV positive | 1 | Not significant(59) |

***CSF:*** *Cerebrospinal Fluid****, HIV****: Human Immune Deficiency Virus, and* ***TB****: Tuberculosis*

**Supplementary file: Table 7:** Narrative synthesis for the risk factors of visual sequelae among patients treated for tuberculosis.

| **Factors** | **Number of studies** | **Direction of association** |
| --- | --- | --- |
| **Participant characteristics** | | |
| Male gender | 3 | Not significant(65-67) |
| Older age | 2 | Risk (65, 66) |
| **Comorbidities** | | |
| Diabetics mellitus | 2 | Not significant(65, 66) |
| Hypertension | 2 | Risk (65) |
|  |  | Not significant(66) |
| Renal disease (non-end stage renal disease (ESRD)) | 1 | Risk (65) |
| Renal disease (ESRD) | 1 | Risk (65) |
| Malignant illness | 1 | Not significant(65) |
| Decrease GFR (ml/min) | 1 | Not significant(66) |
| HIV positive | 1 | Not significant(59) |
| **TB history and treatment** | | |
| Longer duration of Ethambutol (>3months) | 1 | Not significant(65) |
| Daily Ethambutol dose (800-1199) | 1 | Not significant(65) |
| Daily Ethambutol dose (>=1200) | 1 | Not significant(65) |
| Lower ethambutol cumulative dose | 1 | Not significant(66) |
| Longer medication duration | 1 | Risk (66) |
| Miliary TB | 1 | Risk (67) |
| **CNS related characteristics** | | |
| Cranial nerve palsy | 2 | Risk(67, 68) |
| CSF protein >1g/l | 2 | Risk (67) |
|  |  | Not significant(68) |
| Optochiasmatic arachnoiditis in MRI | 1 | Not significant(68) |
| Altered sensorium | 1 | Risk (67) |
| Seizures | 1 | Not significant(67) |
| Hemiparesis | 1 | Not significant(67) |
| Diplopia | 1 | Risk (67) |
| Hydrocephalous | 1 | Not significant(67) |
| Basal exudate | 1 | Risk (67) |
| Infarct | 1 | Not significant(67) |
| Tuberculoma | 1 | Not significant(67) |
| Papilledema | 2 | Risk (67) |
|  |  | Not significant(68) |
| Choroid tubercula | 1 | Not significant(67) |

**Supplementary file: Table 8**: Narrative synthesis for the risk factors of nephrotoxicity and musculoskeletal sequelae among patients treated for tuberculosis.

| **Factors** | **Number of studies** | **Directions of association** |
| --- | --- | --- |
| **Risk factors of nephrotoxicity** | | |
| **Participants characteristics** | | |
| Older age | 2 | Risk (69, 70) |
| Male gender | 1 | Not significant(69) |
| **TB history and treatment** | | |
| KM+TDF compared to KM only | 1 | Not significant(69) |
| KM+ Others compared to KM only | 1 | Risk (69) |
| **Renal related characteristics** | | |
| Acute renal failure | 1 | Risk(70) |
| Positivity of urine TB PCR study | 1 | Not significant(70) |
| Microscopic hematuria | 1 | Not significant(70) |
| Proteinuria | 1 | Not significant(70) |
| **Risk factors of Musculoskeletal sequelae** | | |
| **Participants characteristics** |  |  |
| BMI (>18.5) | 1 | Risk (7) |
| **Comorbidities** | | |
| Chronic kidney disease | 1 | Risk (7) |
| Pre-treatment hyperuricemia | 1 | Risk (7) |

***BMI:*** *Body Mass Index,* ***KM:*** *Kanamycin-based regimen****, PCR:*** *Polymerase Chain Reaction,* ***TDF:*** *Tenofovir Disoproxil Fumarate, and* ***TB****: Tuberculosis*

**Supplementary file Fig 1**: Risk of hearing sequelae stratified by type of TB

**Supplementary file Fig 2**: Risk of acute liver injury stratified by type of TB

**Supplementary file Fig 3:** Risk of renal sequelae stratified by type of TB

**Supplementary file Fig 4**: Risk of lung sequelae stratified by type of TB

Visual sequelae

**Supplementary file Fig 5:** Risk of visual sequelae stratified by type of TB

**Supplementary file Fig 6**: Risk factors of hearing sequelae among DS-TB patients only

**Supplementary file Fig 7**: Risk factors of hearing sequelae among only DR-TB patients

**Supplementary file table 8: Quality assessment for included studies.**

|  | | **Quality of studies for cohort studies** | | | | | | | | | | |
| --- | --- | --- | --- | --- | --- | --- | --- | --- | --- | --- | --- | --- |
|  | |  | | | | | | | | | | |
| **S.NO** | **Study name** | | **Selection** | | | | **Comparability** | **Exposure** | | | **Total** | **Decision** |
|  |  |  | **Adequate case definition** | **Representativeness** | **Selection of controls** | **Definition of controls** | **Comparability of cases and controls** | **Ascertainment of exposure** | **The same method of ascertainment** | **Non-response rate** |  |  |
| 1 | Aggarwal D (1997) | | 0 | 1 | 1 | 1 | 2 | 1 | 1 | 0 | 7 | Moderate |
| 2 | Ali H (2013) | | 0 | 1 | 1 | 1 | 2 | 1 | 0 | 0 | 6 | Moderate |
| 3 | Anand CAC, (2006) | | 1 | 1 | 0 | 0 | 2 | 1 | 1 | 0 | 5 | Moderate |
| 4 | Chang, K.C, 2008) | | 1 | 1 | 1 | 1 | 2 | 1 | 1 | 0 | 8 | High |
| 5 | Chang, K.C, 2007) | | 0 | 1 | 1 | 1 | 1 | 1 | 0 | 0 | 5 | Moderate |
| 6 | Chen H, 2012 | | 0 | 1 | 1 | 1 | 2 | 1 | 1 | 0 | 7 | Moderate |
| 7 | De Lima, 2012 | | 1 | 1 | 1 | 1 | 1 | 1 | 1 | 1 | 9 | High |
| 8 | EJN Pande, 1996 | | 0 | 1 | 1 | 1 | 2 | 0 | 0 | 0 | 5 | Moderate |
| 9 | Ha YJ, 2019 | | 0 | 1 | 1 | 1 | 2 | 0 | 1 | 0 | 6 | Moderate |
| 10 | Kar P, 2018 | | 0 | 1 | 1 | 0 | 1 | 1 | 1 | 0 | 5 | Moderate |
| 11 | Kim EJ, 2018 | | 1 | 1 | 1 | 1 | 2 | 1 | 1 | 0 | 8 | High |
| 12 | Mankhatitham W, 2011 | | 1 | 1 | 1 | 1 | 2 | 1 | 1 | 0 | 8 | High |
| 13 | Mittal S, 2021 | | 1 | 1 | 1 | 1 | 2 | 1 | 1 | 0 | 8 | High |
| 14 | Pasipanodya JG, 2007 | | 1 | 1 | 1 | 1 | 2 | 1 | 1 | 0 | 8 | High |
| 15 | Radovic M, 2016 | | 1 | 1 | 1 | 1 | 2 | 1 | 1 | 1 | 9 | High |
| 16 | Singla R, 2010 | | 1 | 1 | 0 | 1 | 2 | 1 | 1 | 0 | 7 | Moderate |
| 17 | Soemarwoto R., 2021 | | 0 | 0 | 1 | 1 | 2 | 0 | 0 | 0 | 4 | Poor |
| **S.NO** | **Study name** | | **Selection** | | | | **Comparator** | **Outcomes** | | |  |  |
|  |  |  | **Representativeness** | **Selection of non-exposed cohort** | **Ascertainment of exposure** | **Demonstration of outcome is not present at the start** | **Comparability (**) of cohorts on the design or analysis** | **Assessment of outcome** | **Follow-up long enough** | **Adequacy of follow-up** | **Total** | **Decision** |
| 18 | Araujo-Mariz C, (2016) | | 1 | 1 | 1 | 1 | 2 | 1 | 1 | 0 | 8 | High |
| 19 | Auld SC (2021) | | 0 | 0 | 1 | 1 | 2 | 1 | 1 | 1 | 7 | Moderate |
| 20 | Aznar ML(2019) | | 1 | 0 | 1 | 1 | 2 | 1 | 1 | 0 | 7 | Moderate |
| 21 | Bajpai J, 2013 | | 1 | 0 | 1 | 1 | 1 | 1 | 0 | 0 | 5 | Moderate |
| 22 | Candela E, 2003 | | 1 | 1 | 1 | 0 | 2 | 1 | 1 | 0 | 7 | Moderate |
| 23 | Chung K, 2011 | | 1 | 0 | 1 | 0 | 2 | 1 | 1 | 0 | 6 | Moderate |
| 24 | Chushkin MI, 2017 | | 1 | 0 | 1 | 1 | 1 | 1 | 0 | 0 | 5 | Moderate |
| 25 | Cusack RP, 2017 | | 1 | 1 | 0 | 0 | 2 | 1 | 1 | 0 | 6 | Moderate |
| 26 | Ergan B, 2017 | | 1 | 1 | 1 | 0 | 0 | 1 | 1 | 1 | 6 | Moderate |
| 27 | Gupte N, 2019 | | 1 | 0 | 1 | 1 | 2 | 1 | 1 | 1 | 8 | High |
| 28 | Harris T, 2012 | | 1 | 0 | 1 | 1 | 1 | 1 | 0 | 1 | 6 | Moderate |
| 29 | He M, 2023 | | 1 | 0 | 1 | 1 | 0 | 1 | 1 | 0 | 5 | Moderate |
| 30 | Hong H, 2020 | | 1 | 1 | 1 | 0 | 1 | 1 | 1 | 0 | 6 | Moderate |
| 31 | Hoyt KJ, 2019 | | 0 | 0 | 1 | 1 | 2 | 1 | 1 | 1 | 7 | Moderate |
| 32 | Jiang F, 2021 | | 0 | 0 | 1 | 1 | 2 | 1 | 1 | 1 | 7 | Moderate |
| 33 | Jin KW, 2018 | | 0 | 0 | 1 | 0 | 2 | 1 | 1 | 0 | 5 | Moderate |
| 34 | Jo YS, 2017 | | 0 | 1 | 1 | 0 | 2 | 1 | 1 | 0 | 6 | Moderate |
| 35 | Kalita J, 2007 | | 0 | 0 | 1 | 1 | 1 | 1 | 1 | 0 | 5 | Moderate |
| 36 | Khosa C, 2020 | | 0 | 0 | 1 | 1 | 2 | 1 | 1 | 1 | 7 | Moderate |
| 37 | Kwon YS, 2007 | | 0 | 0 | 1 | 0 | 2 | 1 | 1 | 0 | 5 | Moderate |
| 38 | Lee SW, 2010 | | 0 | 0 | 0 | 1 | 2 | 1 | 1 | 0 | 5 | Moderate |
| 39 | Lim J, 2023 | | 0 | 0 | 1 | 1 | 2 | 1 | 1 | 1 | 7 | Moderate |
| 40 | Makhlouf H, 2008 | | 0 | 0 | 1 | 1 | 2 | 1 | 1 | 1 | 7 | Moderate |
| 41 | Marzuki OA, 2008 | | 0 | 0 | 1 | 0 | 2 | 1 | 1 | 1 | 6 | Moderate |
| 42 | Merkler A., 2017 | | 1 | 0 | 1 | 0 | 2 | 1 | 1 | 1 | 7 | Moderate |
| 43 | Perumal, 2018 | | 0 | 0 | 1 | 0 | 2 | 1 | 1 | 1 | 6 | Moderate |
| 44 | Powers M, 2019 | | 0 | 0 | 1 | 1 | 2 | 1 | 1 | 0 | 6 | Moderate |
| 45 | Raj Mani, S, 2021 | | o | 0 | 1 | 0 | 2 | 1 | 1 | 1 | 6 | Moderate |
| 46 | Sagwa EL, 2015 | | 0 | 0 | 1 | 0 | 2 | 1 | 1 | 1 | 6 | Moderate |
| 47 | Saha A, 2016 | | 0 | 0 | 1 | 0 | 2 | 1 | 1 | 0 | 5 | Moderate |
| 48 | Schultz V, 2014 | | 0 | 0 | 1 | 0 | 2 | 1 | 1 | 0 | 5 | Moderate |
| 49 | Seddon J, 2012 | | 0 | 0 | 1 | 0 | 2 | 1 | 1 | 1 | 6 | Moderate |
| 50 | Sharma, 2016 | | 0 | 0 | 1 | 0 | 1 | 1 | 1 | 1 | 5 | Moderate |
| 51 | Shu CC, 2013 | | 0 | 0 | 1 | 0 | 2 | 1 | 1 | 0 | 5 | Moderate |
| 52 | Sinha, 2009 | | 0 | 0 | 1 | 1 | 2 | 1 | 1 | 1 | 7 | Moderate |
| 53 | Sogebi O, 2017 | | 0 | 0 | 1 | 1 | 2 | 1 | 0 | 1 | 6 | Moderate |
| 54 | Tanaviriyachai T, 2023 | | 0 | 0 | 1 | 0 | 2 | 1 | 1 | 1 | 6 | Moderate |
| 55 | Verma R, 2019 | | 0 | 0 | 1 | 1 | 2 | 1 | 1 | 1 | 7 | Moderate |
| 56 | Wang J, 2011 | | 1 | 0 | 1 | 1 | 2 | 1 | 1 | 1 | 8 | High |
| 57 | Wang S, 2018 | | 0 | 0 | 1 | 0 | 2 | 1 | 1 | 0 | 5 | Moderate |
| 58 | Zhao J, 2022 | | 1 | 1 | 1 | 0 | 1 | 1 | 1 | 1 | 7 | Moderate |
| 59 | Zhong T, 2021 | | 1 | 1 | 1 | 0 | 2 | 1 | 1 | 1 | 8 | High |
|  | | **Quality assessment for cross-sectional studies** | | | | | | | | | | |
|  | **Study name** | | **selection** | | | | **Comparators** | **outcome** | | **Total (10%)** | **decision** | |
|  |  | | **Representativeness**  **(*)** | **Sample size (*)** | **Non-respondents (*)** | **Ascertainment of exposure (*)** | **Comparability (**)** | **Assessment of outcome (**)** | **Statistical tests (*)** |  |  |  |
| 60 | Jung JW, 2015 | | 1 | 0 | 0 | 1 | 2 | 1 | 1 | 6 | Moderate | |
| 61 | Park J, 2023 | | 1 | 0 | 0 | 1 | 2 | 2 | 1 | 7 | Moderate | |
| 62 | Lisha P, 2012 | | 1 | 0 | 0 | 1 | 2 | 2 | 1 | 7 | Moderate | |
| 63 | Manji M, 2016 | | 1 | 1 | 1 | 1 | 2 | 1 | 1 | 8 | High | |
| 64 | Mbatchou B, 2016 | | 1 | 1 | 1 | 1 | 2 | 1 | 1 | 8 | High | |
| 65 | Molla Y, 2021 | | 1 | 1 | 1 | 1 | 2 | 2 | 1 | 9 | High | |
| 66 | Mpagama S, 2021 | | 1 | 1 | 1 | 1 | 2 | 2 | 1 | 9 | High | |
| 67 | Namusobya M, 2023 | | 1 | 1 | 1 | 1 | 2 | 2 | 1 | 9 | High | |
| 68 | Nihues S, 2015 | | 0 | 0 | 0 | 1 | 2 | 2 | 1 | 6 | Moderate | |
| 69 | Nkerreuwem S., 2022 | | 0 | 0 | 0 | 1 | 2 | 2 | 1 | 6 | Moderate | |
| 70 | Nuwagira E, 2020 | | 1 | 1 | 1 | 1 | 2 | 1 | 1 | 8 | High | |
| 71 | Popoca-Rodriguze, 2021 | | 0 | 1 | 1 | 1 | 1 | 1 | 1 | 7 | Moderate | |
| 72 | Yimer G, 2008 | | 1 | 1 | 1 | 1 | 2 | 1 | 1 | 8 | High | |
| 73 | Zeleke A, 2020 | | 1 | 1 | 1 | 1 | 2 | 1 | 1 | 8 | High | |

**Supplementary file:** Publication bias

Six statistically significant variables for lung sequelae were assessed for possible publication bias using funnel plots and Egger’s regression test. The Egger regression test showed that publication bias was not significant in any of these variables: older age (P-value=0.5183), history of smoking (P-value=0.6779), previous TB treatment (p= 0.4025), smear positive (P-value=0.7440), alcohol drinkers (p=0.9601), and presence of pulmonary lesions (P-value=0.2240) **(Figures 7 and 12)**.

Five statistically significant variables for hepatotoxicity were identified, and the Egger regression test showed there to be no publication bias for these: CD4 count <200mm^3^ (P-value=0.5486), existing hepatitis (P-value=0.3250), previous TB treatment history (P-value=0.7327), hypo-albuminemia (P-value=0.4466), and HIV co-infection (P-value=0.6991) **(Figures 13 and 17)**. The Egger regression test also revealed non-significant findings for baseline hearing problems (P-value=0.9591) and HIV co-infection (p=0.5064), two variables that were found to be significantly associated with hearing sequelae **(Figures 18 and 19).**

**Figure 7:** Forrest plot for older age in studies investigating lung sequelae.

**Figure 8:** Funnel plot for smoking status in studies investigating lung sequelae.

**Figure 9:** Funnel plot for previous TB treatment hearing sequelae among the included studies.

**Figure 10:** Funnel plot for smear positive in studies investigating lung sequelae.

**Figure 11:** Funnel plot for alcohol drinkers in studies investigating lung sequelae.

**Figure 12:** Funnel plot for presence of pulmonary lesions in studies investigating lung sequelae.

**Figure 13:** Funnel plot for CD4 <200mm^3^ in studies investigating hepatotoxicity sequelae.

**Figure 14:** Funnel plot for existing chronic hepatitis in studies investigating hepatotoxicity.

**Figure 15:** Funnel plot for previous TB in studies investigating hepatotoxicity.

**Figure 16:** Funnel plot for hypo-albuminemia in studies investigating hepatotoxicity.

**Figure 17:** Funnel plot for HIV status in studies investigating hepatotoxicity.

**Figure 18:** Funnel plot for baseline hearing loss in studies investigating hearing loss.

**Figure 19:** Funnel plot for baseline hearing loss in studies investigating hearing loss.

**Supplementary file:** Galbraith plots for precision and heterogeneity

Galbraith plots were used to evaluate the precision and heterogeneity amongst studies analyzed for respiratory, hepatic, and hearing sequelae. The horizontal axis represents 1/standard error while the Y-axis represents the effect or standard error. The red line represents the regression line, the green line shows the effect, and the gray shadow represents 95% CI. The plots show there to be no heterogeneity, as over 95% of studies (points) are between the 95% CI limits **(Figures 20-32)**.

**Figure 20:** Galbraith plot for older age in studies investigating lung sequelae.

**Figure 21:** Galbraith plot for smoking status in studies investigating lung sequelae.

**Figure 22:** Galbraith plot for previous TB treatment in studies investigating lung sequelae.

**Figure 23:** Galbraith plot for smear positive status in studies investigating lung sequelae.

**Figure 24:** Galbraith plot for alcohol drinkers in studies investigating lung sequelae.

**Figure 25:** Galbraith plot for the presence of pulmonary lesions in studies investigating lung sequelae.

**Figure 26:** Galbraith plot for CD4 <200mm^3^ in studies investigating hepatotoxicity sequelae.

**Figure 27:** Galbraith plot for pre-existing chronic hepatitis in studies investigating hepatotoxicity.

**Figure 28:** Galbraith plot for previous TB in studies investigating hepatotoxicity.

**Figure 29:** Galbraith plot for hypo-albuminemia in studies investigating hepatotoxicity.

**Figure 30:** Galbraith plot for HIV status in studies investigating hepatotoxicity.

**Figure 31:** Galbraith plot for baseline hearing problem in studies investigating hearing loss.

**Figure 32:** Galbraith plot for HIV status in studies investigating hearing loss.

**References**

1. Aggarwal D, Gupta A, Janmeja AK, Bhardwaj M. Evaluation of tuberculosis-associated chronic obstructive pulmonary disease at a tertiary care hospital: A case-control study. Lung India. 2017;34(5):415-9.

2. Chushkin MI, Ots ON. Impaired pulmonary function after treatment for tuberculosis: the end of the disease? Jornal brasileiro de pneumologia : publicacao oficial da Sociedade Brasileira de Pneumologia e Tisilogia. 2017;43(1):38-43.

3. Manji M, Shayo G, Mamuya S, Mpembeni R, Jusabani A, Mugusi F. Lung functions among patients with pulmonary tuberculosis in Dar es Salaam - a cross-sectional study. BMC Pulmonary Medicine. 2023;16.

4. Mpagama SG, Msaji KS, Kaswaga O, Zurba LJ, Mbelele PM, Allwood BW, et al. The burden and determinants of post-TB lung disease. The international journal of tuberculosis and lung disease : the official journal of the International Union against Tuberculosis and Lung Disease. 2021;25(10):846-53.

5. Powers M, Sanchez TR, Welty TK, Cole SA, Oelsner EC, Yeh F, et al. Lung Function and Respiratory Symptoms after Tuberculosis in an American Indian Population. The Strong Heart Study. Annals of the American Thoracic Society. 2020;17(1):38-48.

6. Gupte AN, Paradkar M, ar, Selvaraju S, Kannan T, Shri Vijay Bala Yogendra S, et al. Assessment of lung function in successfully treated tuberculosis reveals high burden of ventilatory defects and COPD. PLoS One. 2023;14(5).

7. Ha YJ, Chung SW, Lee JH, Kang EH, Lee YJ, Song YW. Clinical features and risk factors for gout attacks during anti-tuberculosis treatment: A case-control study in South Korea. International Journal of Rheumatic Diseases. 2019;22(10):1905-11.

8. Jo YS, Park J-H, Lee JK, Heo EY, Chung HS, Kim DK. Risk factors for pulmonary arterial hypertension in patients with tuberculosis-destroyed lungs and their clinical characteristics compared with patients with chronic obstructive pulmonary disease. International journal of chronic obstructive pulmonary disease. 2017;12:2433-43.

9. Khosa C, Bhatt N, Massango I, Azam K, Saathoff E, Bakuli A, et al. Development of chronic lung impairment in Mozambican TB patients and associated risks. BMC pulmonary medicine. 2020;20(1):127.

10. Lee SW, Kim YS, Kim DS, Oh YM, Lee SD. The risk of obstructive lung disease by previous pulmonary tuberculosis in a country with intermediate burden of tuberculosis. Journal of Korean Medical Science. 2011;26(2):268-73.

11. Lisha PV, James Pt Fau - Ravindran C, Ravindran C. Morbidity and mortality at five years after initiating Category I treatment among patients with new sputum smear positive pulmonary tuberculosis. (0019-5707 (Print)).

12. Nkereuwem E, Agbla S, Sallahdeen A, Owolabi O, Sillah AK, Genekah M, et al. Reduced lung function and health-related quality of life after treatment for pulmonary tuberculosis in Gambian children: a cross-sectional comparative study. Thorax. 2023.

13. Auld SC, Kornfeld H, Maenetje P, Mlotshwa M, la, Chase W, et al. Pulmonary restriction predicts long-term pulmonary impairment in people with HIV and tuberculosis. BMC pulmonary medicine. 2021;21(1):19.

14. Bajpai J, Kant S, Verma A, Bajaj DK. Clinical, Radiological, and Lung Function Characteristics of Post-tuberculosis Bronchiectasis: An Experience From a Tertiary Care Center in India. Cureus. 2023;15(2).

15. Jae-Woo J, Choi J-C, Jong-Wook S, Kim J-Y, Choi B-W, Park I-W. Pulmonary Impairment in Tuberculosis Survivors: The Korean National Health and Nutrition Examination Survey 2008-2012. PLoS One. 2023;10(10).

16. Nuwagira E, Stadelman A, Baluku JB, Rhein J, Byakika-Kibwika P, Mayanja H, et al. Obstructive lung disease and quality of life after cure of multi-drug-resistant tuberculosis in Uganda: A cross-sectional study. Tropical Medicine and Health. 2020;48(1):34.

17. Pasipanodya JG, Miller TL, Vecino M, Munguia G, Garmon R, Bae S, et al. Pulmonary impairment after tuberculosis. Chest. 2007;131(6):1817-24.

18. Hoyt KJ, ⨯ Sonali S, White L, Noyal MJ, Salgame P, Lakshminarayanan S, et al. Effect of malnutrition on radiographic findings and mycobacterial burden in pulmonary tuberculosis. PLoS One. 2023;14(3).

19. Soemarwoto RAS, Mesah AD, Rusmini H, Arlek M. Factors affecting the occurrence of tuberculosis destroyed lung. Indian Journal of Forensic Medicine and Toxicology. 2021;15(1):1432-7.

20. Lee J, Park HJ, Byun MK, Kim CY, Shin S, Kim Y, et al. Airflow obstruction and chronic obstructive pulmonary disease in pulmonary tuberculosis. Respirology. 2023;28:273-4.

21. Nihues Sde S, Mancuzo EV, Sulmonetti N, Sacchi FP, Viana Vde S, Netto EM, et al. Chronic symptoms and pulmonary dysfunction in post-tuberculosis Brazilian patients. (1678-4391 (Electronic)).

22. Mbatchou Ngahane B, Hugo, Nouyep J, Ng, a Motto M, Mapoure Njankouo Y, et al. Post-tuberculous lung function impairment in a tuberculosis reference clinic in Cameroon. Respiratory medicine. 2016;114:67-71.

23. He M, Yang X, Zhang Z, Liu Z. Impaired pulmonary function and associated factors in the elderly with tuberculosis on admission: a preliminary report. BMC Infectious Diseases. 2023;23:1-8.

24. Chung K-P, Chen J-Y, Lee C-H, Wu H-D, Wang J-Y, Lee L-N, et al. Clinics (Sao Paulo, Brazil). 2011;66(4):549-56.

25. Radovic M, Ristic L, Ciric Z, Dinic-Radovic V, Stankovic I, Pejcic T, et al. Changes in respiratory function impairment following the treatment of severe pulmonary tuberculosis - limitations for the underlying COPD detection. International Journal of COPD. 2016;11(1):1307-16.

26. Namusobya M, Bongomin F, John M, Kimuli I, Ddungu A, Batte C, et al. Chronic respiratory symptoms and chronic obstructive pulmonary disease following completion of pulmonary tuberculosis treatment in Uganda. medRxiv. 2023.

27. ela A, Andujar J, Hernández L, Martín C, Barroso E, Arriero JM, et al. Functional sequelae of tuberculous pleurisy in patients correctly treated. Chest. 2003;123(6):1996-2000.

28. An, AC, Seth AK, Paul M, Puri P. Risk factors of hepatotoxicity during anti-tuberculosis treatment. Medical Journal Armed Forces India. 2006;62(1):45-9.

29. Zhong T, Fan Y, Dong XL, Guo X, Wong KH, Wong WT, et al. An Investigation of the Risk Factors Associated With Anti-Tuberculosis Drug-Induced Liver Injury or Abnormal Liver Functioning in 757 Patients With Pulmonary Tuberculosis. Frontiers in Pharmacology. 2021;12.

30. Shu CC, Lee CH, Lee MC, Wang JY, Yu CJ, Lee LN. Hepatotoxicity due to first-line anti-tuberculosis drugs: A five-year experience in a Taiwan medical centre. International Journal of Tuberculosis and Lung Disease. 2013;17(7):934-9.

31. Araújo-Mariz C, Edmundo Pessoa L, Acioli-Santos B, Maruza M, Ulisses Ramos M, Ricardo Arraes de Alencar X, et al. Hepatotoxicity during Treatment for Tuberculosis in People Living with HIV/AIDS. PLoS One. 2023;11(6).

32. Aznar ML, Rando Segura A, Moreno MM, Espasa M, Sulleiro E, Bocanegra C, et al. Treatment Outcomes and Adverse Events from a Standardized Multidrug-Resistant Tuberculosis Regimen in a Rural Setting in Angola. (1476-1645 (Electronic)).

33. Cusack R, Chawke L, O'Brien D, O'Connor T. Predictors of hepatotoxicity among patients with tuberculosis treated with antituberculous medication. European Respiratory Journal. 2016;48.

34. de Lima MFS, de Melo HRL. Hepatotoxicity induced by antituberculosis drugs among patients coinfected with HIV and tuberculosis. Cadernos de Saude Publica. 2012;28(4):698-708.

35. ERGAN B, Kirmizigul E, Uzun Ö, Çöplü L. Risk factors for hepatotoxicity in patients hospitalized for tuberculosis. European Journal of General Medicine. 2017;14(1).

36. Kwon YS, Koh W-J, Suh GY, Chung MP, Kim H, Kwon OJ. Hepatitis C virus infection and hepatotoxicity during antituberculosis chemotherapy. Chest. 2007;131(3):803-8.

37. Lim J, Kim JS, Kim HW, Kim YH, Jung SS, Kim JW, et al. Metabolic Disorders Are Associated with Drug-Induced Liver Injury during Antituberculosis Treatment: A Multicenter Prospective Observational Cohort Study in Korea. Open Forum Infectious Diseases. 2023;10(8).

38. Mankhatitham W, Lueangniyomkul A, Manosuthi W. Hepatotoxicity in patients co-infected with tuberculosis and HIV-1 while receiving non-nucleoside reverse transcriptase inhibitor-based antiretroviral therapy and rifampicin-containing anti-tuberculosis regimen. Southeast Asian Journal of Tropical Medicine and Public Health. 2011;42(3):651-8.

39. Raj Mani S, Iyyadurai R, Mishra A, Manjunath K, Prasad J, Lakshmanan J, et al. Predicting antitubercular drug-induced liver injury and its outcome and introducing a novel scoring system. International Journal of Mycobacteriology. 2021;10(2):116-21.

40. Molla Y, Wubetu M, Bekalu D. Anti-Tuberculosis Drug Induced Hepatotoxicity and Associated Factors among Tuberculosis Patients at Selected Hospitals, Ethiopia. Hepatic Medicine : Evidence and Research. 2023;13:1-8.

41. Alima Hassen A, Tefera B, Yami A, Ayen WY. Anti-Tuberculosis Drug Induced Hepatotoxicity among TB/HIV Co-Infected Patients at Jimma University Hospital, Ethiopia: Nested Case-Control Study. PLoS One. 2023;8(5).

42. Zeleke A, Misiker B, Yesuf TA. Drug-induced hepatotoxicity among TB/HIV co-infected patients in a referral hospital, Ethiopia. BMC research notes. 2020;13(1):2.

43. Makhlouf HA, Helmy A, Fawzy E, El-Attar M, Rashed HAG. A prospective study of antituberculous drug-induced hepatotoxicity in an area endemic for liver diseases. Hepatology International. 2008;2(3):353-60.

44. Chang KC, Leung CC, Yew WW, Tam CM. Standard anti-tuberculosis treatment and hepatotoxicity: Do dosing schedules matter? European Respiratory Journal. 2007;29(2):347-51.

45. Saha A, Shanthi F X M, Winston A B, Das S, Kumar A, Michael JS, et al. Prevalence of Hepatotoxicity From Antituberculosis Therapy: A Five-Year Experience From South India. Journal of primary care & community health. 2016;7(3):171-4.

46. Yimer G, Aderaye G, Amogne W, Makonnen E, Aklillu E, Lindquist L, et al. Anti-tuberculosis therapy-induced hepatotoxicity among Ethiopian HIV-positive and negative patients. PLoS ONE. 2008;3(3):e1809.

47. Marzuki OA, Fauzi ARM, Ayoub S, Kamarul Imran M. Prevalence and risk factors of anti-tuberculosis drug-induced hepatitis in Malaysia. Singapore Medical Journal. 2008;49(9):688-93.

48. Kar P, Karna R, Ruttala R, Arora S, Chakravarty A, Kumar S. Clinical and Molecular Risk Factors of Anti-tubercular Therapy Induced Hepatitis. Journal of Clinical and Experimental Hepatology. 2019;9(2):200-6.

49. Wang S, Shangguan Y, Ding C, Li P, Ji Z, Shao J, et al. Risk factors for acute liver failure among inpatients with anti-tuberculosis drug-induced liver injury. Journal of International Medical Research. 2020;48(1).

50. e JN, Singh SPN, Khilnani GC, Khilnani S, on RK. Risk factors for hepatotoxicity from antituberculosis drugs: A case-control study. Thorax. 1996;51(2):132-6.

51. Schultz V, Marroni CA, Amorim CS, Baethgen LF, Pasqualotto AC. Risk factors for hepatotoxicity in solid organ transplants recipients being treated for tuberculosis. Transplantation Proceedings. 2014;46(10):3606-10.

52. Wang JY, Liu CH, Hu FC, Chang HC, Liu JL, Chen JM, et al. Risk factors of hepatitis during Anti-tuberculous treatment and implications of hepatitis virus load. Journal of Infection. 2011;62(6):448-55.

53. Singla R, Sharma SK, Mohan A, Makharia G, Sreenivas V, Jha B, et al. Evaluation of risk factors for antituberculosis treatment induced hepatotoxicity. The Indian journal of medical research. 2010;132:81-6.

54. Sagwa EL, Ruswa N, Mavhunga F, Rennie T, Leufkens HGM, Mantel-Teeuwisse AK. Comparing amikacin and kanamycin-induced hearing loss in multidrug-resistant tuberculosis treatment under programmatic conditions in a Namibian retrospective cohort. BMC Pharmacology and Toxicology. 2015;16(1):36.

55. Seddon JA, Thee S, Jacobs K, Ebrahim A, Hesseling AC, Schaaf HS. Hearing loss in children treated for multidrug-resistant tuberculosis. Journal of Infection. 2013;66(4):320-9.

56. Sogebi OA, Adefuye BO, Adebola SO, Oladeji SM, Adedeji TO. Clinical predictors of aminoglycoside-induced ototoxicity in drug-resistant Tuberculosis patients on intensive therapy. Auris Nasus Larynx. 2017;44(4):404-10.

57. Hong H, Dowdy DW, Dooley KE, Francis HW, Budhathoki C, Han HR, et al. Risk of hearing loss among multidrug-resistant tuberculosis patients according to cumulative aminoglycoside dose. International Journal of Tuberculosis and Lung Disease. 2020;24(1):65-72.

58. Harris T, Bardien S, Schaaf HS, Petersen L, De Jong G, Fagan JJ. Aminoglycoside-induced hearing loss in HIV-positive and HIV-negative multidrug-resistant tuberculosis patients. South African medical journal = Suid-Afrikaanse tydskrif vir geneeskunde. 2012;102(6):363-6.

59. Merkler A, E. e, Reynolds A, S. r, Gialdini G, Morris NA, et al. Neurological complications after tuberculous meningitis in a multi-state cohort in the United States. Journal of the neurological sciences. 2017;375:460-3.

60. Sharma V, Bhagat S, Verma B, Singh R, Singh S. Audiological evaluation of patients taking Kanamycin for multidrug resistant tuberculosis. Iranian Journal of Otorhinolaryngology. 2016;28(3):203-8.

61. Tanaviriyachai T, Choovongkomol K, Pornsopanakorn P, Jongkittanakul S, Piyapromdee U, Sudprasert W. Factors Affecting Neurological Deficits in Thoracic Tuberculous Spondylodiscitis. International Journal of Spine Surgery. 2023;17(5):645-51.

62. Mittal S, Yadav G, eep, Ahuja K, Ifthekar S, Sarkar B, et al. Predicting neurological deficit in patients with spinal tuberculosis – A single-center retrospective case-control study. SICOT-J. 2022;7.

63. Zhao J, Cai Z, Meng Y, Zhou X, Jiang H. Predictive Factors for Late-Onset Neurological Deficits in Patients with Posttuberculous Thoracic Kyphosis. BioMed research international. 2022;2022:8555924.

64. Kalita J, Misra UK, Ranjan P. Predictors of long-term neurological sequelae of tuberculous meningitis: a multivariate analysis. European journal of neurology. 2007;14(1):33-7.

65. Chen HY, Lai SW, Muo CH, Chen PC, Wang IJ. Ethambutol-induced optic neuropathy: A nationwide population-based study from Taiwan. British Journal of Ophthalmology. 2012;96(11):1368-71.

66. Jin KW, Lee JY, Rhiu S, Choi DG. Longitudinal evaluation of visual function and structure for detection of subclinical Ethambutol-induced optic neuropathy. PLoS ONE. 2019;14(4).

67. Verma R, Sarkar S, Garg RK, Malhotra HS, Sharma PK, Saxena S. Ophthalmological manifestation in patients of tuberculous meningitis. QJM : monthly journal of the Association of Physicians. 2019;112(6):409-19.

68. Sinha MK, Garg RK, Agarwal A, Singh MK, Verma R, Shukla R. Vision impairment in tuberculous meningitis: Predictors and prognosis. Journal of the Neurological Sciences. 2010;290(1):27-32.

69. Perumal R, Abdelghani N, Naidu N, Yende-Zuma N, Dawood H, Naidoo K, et al. Risk of nephrotoxicity in patients with drug-resistant tuberculosis treated with Kanamycin/capreomycin with or without concomitant use of tenofovir-containing antiretroviral therapy. Journal of Acquired Immune Deficiency Syndromes. 2018;78(5):536-42.

70. Kim EJ, Lee W, Jeong WY, Choi H, Jung IY, Ahn JY, et al. Chronic kidney disease with genitourinary tuberculosis: old disease but ongoing complication. BMC nephrology. 2018;19(1):193.
