# Supplementary material for: Risk factors associated with post-tuberculosis sequelae: a systematic review and meta-analysis
Source: eClinicalMedicine. 2024 Oct 21;77:102898. doi: 10.1016/j.eclinm.2024.102898 (PMC11535315; doi:10.1016/j.eclinm.2024.102898)
Supplement: Translated abstract [file mmc2.docx]

The following Amharic translations were provided by the authors and are reproduced as submitted. These translations have not undergone peer review. Our editorial processes have only been applied to the original English abstract, which should be considered the reference for this manuscript.

**ማጠቃለያ**

**መግቢያ፦** ከሳንባ ነቀርሳ (ቲቢ) በሽታ ህክምና በኋላ ከበሽታው የዳኑ ታካሚዎች ለተለያዩ ከህክምናው በኋላ ለሚመጡ የጤና ችግሮች ይጋለጣሉ:: ከሳንባ ነቀርሳ ህክምና በኋላ የሚመጣን የጤና እክል መንስኤ መለየት ከበሽታው የዳኑ ሰዎችን ጤንነት ለመጠበቅ እና ጤናማ ህይወት እንዲኖሩ ለማድረግ አስፈላጊ ነው። የዚህ ጥናት ዋና ዓላማ ከዚህ በፊት የታተሙ የምርምር ስራዎችን ስልታዊ በሆነ መንገድ በመለየትና በመተንተን ለድኅረ የሳንባ ነቀርሳ የጤና ችግር የሚዳርጉ መንስኤዎችን መለየት ነው::

**የምርምር ዘዴዎች፦** ለሳንባ ነቀርሳ ታክመው ከዳኑ ሰዎች ላይ በሚከሰት የአካላዊ የጤና ጉዳቶች ላይ እስከ ታህሳስ 12፣ 2023 እ.አ.አ ድረስ የታተሙ የምርምር ስራዎችን ከሜድላይን ፣ ኢምቤዝ ፣ ፕሮኩዌስትና እና ስኮፐስ የመረጃ ቋጦች ስልታዊ በሆነ መንገድ እንዲካተቱ አድርገናል፡፡ የእነዚህም ጥናቶች የጥናታቸው ዋና ውጤት ለረጅም ጊዜ የአካል ጉዳት (ማለትም የመተንፈሻ አካላት፣ የጉበት፣ የመስማት፣ የነርቭ፣ የእይታ፣ የኩላሊት ፣ የጡንቻ እና አጽም እክል) የሚዳርጉ መንስኤዎችን መለየት ነበር፡፡

ለረጅም ጊዜ የሚዘልቁ አካላዊ ድኅረ ህክምና ችግሮች ያጋጠሟቸውን የሳንባ ነቀርሳ ሕሙማን በሙሉ በጥናቱ አካተናል። የዕድል አሃዝን (ዕአ) እና 95% በራስ የመተማመን ጊዜን (በየጊ) ተጠቅመን የጥናቱን ግኝቶች ተርጉመናል። የህትመት ወገናዊነት (ፐፕሊኬሽን ቢያዝ) በኤግር ሬግሬሽን አማካኝነት መርምረናል። ፕሮቶኮሉ በፕሮስፖሮ (CRD42021250909) ላይ ተመዝግቧል ።

**ግኝቶች**፦ በዚህ ጥናት ውስጥ ለረጅም ጊዜ የሚዘልቁ አካላዊ ተጓዳኝ ችግሮች ያጋጠሟቸውን 31,553 የሳንባ ነቀርሳ ሕሙማንን ከ 28 ሀገራት ከተወጣጡ 73 ጥናቶች አካተናል፡፡ የሳምባ ነቀርሳ በሽታ በኋላ የመተንፈሻ አካላት ችግሮችን የሚያስከትሉ ዋነኛ ምክንያቶች በዕድሜ መግፋት (ዕአ=1.62, 95% በየጊ 1.07-2.47) ፣ከዚህ በፊት የቲቢ ህክምና ታሪክ (ዕአ=3.43, 95% በየጊ 2.37-4.97) ፣ ማጨስ (ዕአ=1.41, 95% በየጊ 1.09-1.83) ፣ የአልኮል መጠጥ መጠጣት (ዕአ=1.84, 95% በየጊ 1.04-3.25) ፣ በአክታ ውስጥ የሳንባ ነቀርሳ ባክቴራያ መኖር (ዕአ=3.11, 95% በየጊ 1.77-6.44) እና ሕክምና በተጀመረበት ጊዜ በራጅ ውስጥ ቁስለቶች መታየት (ዕአ=2.04, 95% በየጊ 1.07-3.87) ናቸው፡፡ ከቲቢ በኋላ በጉበት ላይ ከሚደርሰው ጉዳት ጋር ተያይዘው የሚመጡ አደጋዎች ዋነኛ ምክንያቶች; የጉበት ቫይረስ (ዕአ=2.41, 95% በየጊ 1.16-6.08) ፣ ከዚህ በፊት የቲቢ ህክምና (ዕአ=2.64, 95% በየጊ 1.22-6.67) ፣ ዝቅተኛ የአልቡሚን መጠን (ዕአ=2.10, 95% በየጊ 1.53-2.88) ፣ ኤችአይቪ በሽታ (ዕአ=2.72, 95% በየጊ 1.66-4.46) እና በደም ውስጥ ያለ የሲዲ4 መጠን <200 mm3 (ዕአ=2.03, 95% በየጊ 1.26-3.27) ናቸው። ከቲቢ የመስማት ችግር ጋር ተያይዘው የሚመጡ የአካላዊ የጤና ጉዳቶች ዋነኛ ምክንያቶች ደግሞ መሠረታዊ የመስማት ችግሮች (ዕአ=1.72, 95% በየጊ 1.30-2.26) እና የኤችአይቪ በደም ውስጥ መኖር (ዕአ=3.02, 95% በየጊ 1.96-4.64) ናቸው።

**ትርጉም**፦ ይህ ሥርዓት ባለው መንገድ የተደረገ ምርመር ለረጅም ጊዜ የሚቆዩ አካላዊ ተጓዳኝ ችግሮች ማለትም የመተንፈሻ አካላት፣ የጉበት እንዲሁም የመስማት ችግሮችከተለያዩ የማህበራዊ፣የዴሞግራፊያዊ፣ የባሕርይና የሕክምና (ከክሊኒካዊ) ምክንያቶች ጋር የተያያዙ መሆናቸውን መለየት ችሏል ። እነዚህን አደገኛ መንስኤ ምክንያቶችን ለይቶ ማወቅ ከቲቢ ሕክምና በኋላ የሚደርሰውን ሥቃይ ለመቀነስ ይረዳል።

**የገንዘብ ድጋፍ** -ሂሊ ሜዲካል ሪሰርች ሬይን ፋውንዴሽን, የአውስትራሊያ ብሔራዊ የጤና እና ህክምና ምርምር ምክር ቤት እና የከርቲን ዩኒቨርሲቲ የከፍተኛ ዲግሪ ምርምር ስኮላርሺፕ ለጥናቱ ድጋፍ አድርገዋል፡፡

**ቁልፍ ቃላት:-** የሳንባ ነቀርሳ ፣ አካላዊ ተጓዳኝ ችግሮች ፣ ሥርዓታዊ ክለሳ
